# A minimal human physiologically based kinetic model of thyroid hormones and chemical disruption of plasma thyroid hormone binding proteins

**DOI:** 10.3389/fendo.2023.1168663

**Published:** 2023-05-25

**Authors:** Anish D. Bagga, Brian P. Johnson, Qiang Zhang

**Affiliations:** ^1^ Emory College of Arts and Sciences, Emory University, Atlanta, GA, United States; ^2^ Department of Pharmacology and Toxicology, Michigan State University, East Lansing, MI, United States; ^3^ Gangarosa Department of Environmental Health, Rollins School of Public Health, Emory University, GA, Atlanta, United States

**Keywords:** thyroid hormone, thyroid hormone binding protein, thyroxine-binding globulin, transthyretin, albumin, PBK model, tissue delivery, endocrine disrupting chemicals

## Abstract

The thyroid hormones (THs), thyroxine (T4) and triiodothyronine (T3), are under homeostatic control by the hypothalamic-pituitary-thyroid axis and plasma TH binding proteins (THBPs), including thyroxine-binding globulin (TBG), transthyretin (TTR), and albumin (ALB). THBPs buffer free THs against transient perturbations and distribute THs to tissues. TH binding to THBPs can be perturbed by structurally similar endocrine-disrupting chemicals (EDCs), yet their impact on circulating THs and health risks are unclear. In the present study, we constructed a human physiologically based kinetic (PBK) model of THs and explored the potential effects of THBP-binding EDCs. The model describes the production, distribution, and metabolism of T4 and T3 in the *Body Blood*, *Thyroid*, *Liver*, and *Rest-of-Body (RB)* compartments, with explicit consideration of the reversible binding between plasma THs and THBPs. Rigorously parameterized based on literature data, the model recapitulates key quantitative TH kinetic characteristics, including free, THBP-bound, and total T4 and T3 concentrations, TH productions, distributions, metabolisms, clearance, and half-lives. Moreover, the model produces several novel findings. (1) The blood-tissue TH exchanges are fast and nearly at equilibrium especially for T4, providing intrinsic robustness against local metabolic perturbations. (2) Tissue influx is limiting for transient tissue uptake of THs when THBPs are present. (3) Continuous exposure to THBP-binding EDCs does not alter the steady-state levels of THs, while intermittent daily exposure to rapidly metabolized TBG-binding EDCs can cause much greater disruptions to plasma and tissue THs. In summary, the PBK model provides novel insights into TH kinetics and the homeostatic roles of THBPs against thyroid disrupting chemicals.

## 1 Introduction

Thyroxine (T4) and triiodothyronine (T3) are thyroid hormones (THs) that regulate several vital physiological processes: growth, differentiation, development of the nervous system, cardiovascular function, reproduction, and energy metabolism (1–5). The circulating TH levels are under robust homeostatic control to keep within narrow physiological ranges. Structured as a negative feedback loop, the hypothalamic-pituitary-thyroid (HPT) axis is the primary homeostatic mechanism. The thyrotropin-releasing hormone (TRH) produced by neurons in the hypothalamic paraventricular nucleus (PVN) stimulates the synthesis and release of the thyroid-stimulating hormone (TSH) in the anterior pituitary. TSH then stimulates the production and release of T4 and T3 by the thyroid follicles (6). Negative feedback from T4 and T3 occurs through the inhibition of both TRH and TSH, which keeps the circulating free TH levels close to the setpoints in the long term. In contrast, a separate mechanism is in place to ensure TH homeostasis against transient perturbations, which involves three major TH binding/distributor proteins (THBPs/THDPs) in the blood: thyroxine-binding globulin (TBG), transthyretin (TTR), and albumin (ALB). Overall, greater than 99.97% of plasma T4 is bound to THBPs, while about 99.7% of plasma T3 is bound to THBPs (7–9). The majority of the THBP molecules exist unoccupied by THs (9, 10). The unique concentration ratios of T4, T3, THBPs, and their complexes (TH-THBPs) generate a strong buffer system where transient changes of free T4 and T3 can be quickly compensated by the absorption or release of T4 and T3 by the THBPs. With high amounts of THs stored in THBPs, TH-THBPs also act as a reservoir in the event of short-term deficiency of THs. Moreover, THBPs are required to maintain a uniform delivery of circulating THs throughout the perfused tissues (11, 12).

A variety of environmental chemicals have structures similar to THs and can interfere with TH binding to THBPs. These endocrine-disrupting chemicals (EDCs) include polychlorinated biphenyls (PCBs), dibenzo-p-dioxins, dibenzofurans, and brominated flame retardants such as polybrominated diphenyl ethers (PBDEs), pentabromophenol (PBP), and tetrabromobisphenol A (TBBPA) (13–18). Some of them such as certain PBDEs even have higher binding affinities for TBG or TTR than the endogenous hormones (18, 19). It has been suspected that these EDCs can displace THs from THBPs, causing an increase in free plasma THs (14, 20). However, given the fact that the predominant fractions of THBPs are unoccupied by THs (thus available for EDC binding) and the cross-buffering effect between THBPs, it is unlikely that the free THs will be altered tangibly by THBP-binding EDCs. The effect size of these thyroid disruptors is not well characterized and a significant knowledge gap exists.

Mathematical modeling has been used to understand the various aspects of the thyroid system and its responses to dietary, therapeutic, and environmental perturbations (21–29). A physiologically based kinetic (PBK) model of THs containing sufficient details such as all the THBP binding events will help to provide critical insights to the EDC effects. Existing TH kinetic models are insufficient for our purpose here because none of them have all the necessary model components to address these questions (21–24, 26, 27, 29). Here we constructed a minimally sufficient human PBK model of THs and used the model to investigate the effects of THBP-binding EDCs. We started by formulating a multicompartment model to include TH production, distribution, and metabolism. The model contains the thyroid, liver, and rest-of-body compartments with explicit descriptions of the reversible binding kinetics between THs and THBPs in the blood compartments. The model was parameterized and validated extensively based on literature data. Here we reported several novel findings on TH kinetics and the effects of EDCs.

## 2 Methods

### 2.1 Construction of the human PBK model

#### 2.1.1 Model structure

**Figure 1:**
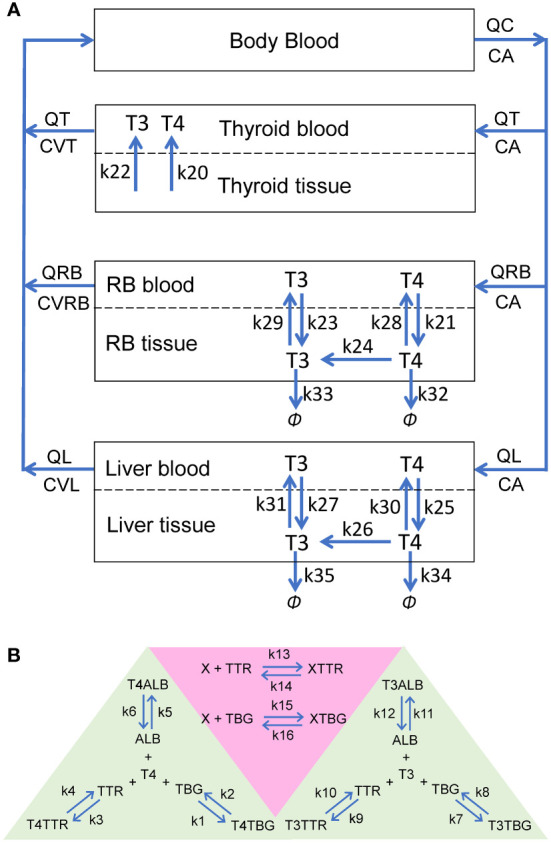
Schematic illustrations of the human PBK model for T4 and T3. **(A)** The overall structure of the PBK model. *RB*: rest-of-body. **(B)** Reversible binding of T4 and T3 with TBG, TTR and ALB (green-shaded area), and reversible binding of an EDC, *X*, with TBG or TTR (pink-shaded area). These binding events occur in the plasma of all blood compartments.

The PBK model contains four overall compartments: *Body Blood*, *Thyroid*, *Liver*, and *Rest-of-Body* (*RB*) (**Figure 1A**). The *Liver*, *RB*, and *Thyroid* compartments are further divided into tissue blood (vasculature) and tissue proper (extra-vasculature) subcompartments. Since we are primarily concerned with the steady-state TH behaviors in the present study, the negative feedback regulation of TSH by THs is not considered. The potential limitations in this regard are addressed in Discussion.

#### 2.1.2 Kinetic processes

In each blood (sub)compartments, there are 11 molecular species (state variables): free T4 (*fT4*), T4-bound TBG (*T4TBG*), T4-bound TTR (*T4TTR*), T4-bound ALB (*T4ALB*), free T3 (*fT3*), T3-bound TBG (*T3TBG*), T3-bound TTR (*T3TTR*), T3-bound ALB (*T3ALB*), free TBG (*TBG*), free TTR (*TTR*), and free ALB (*ALB*) (**Figure 1B**, green-shaded area). Each blood (sub)compartment is assumed to be well-mixed and has its own set of ordinary differential equations (ODEs) tracking the (sub)compartment-specific rates of change of these variables. The interaction between each TH and each THBP species follows the law of mass action, which involves a reversible binding process: second-order association and first-order dissociation. For each specific interaction, its binding kinetic constants (i.e., the association rate constant and dissociation rate constant) are assumed the same across all blood (sub)compartments.

In the *Body Blood* compartment, in addition to the binding events between THs and THBPs, the venous blood inflows coming back from the *Thyroid*, *Liver*, and *RB* compartments and arterial blood outflow are also described for each of the 11 molecular species. Similarly, these species enter each tissue blood subcompartment through the corresponding arterial blood inflow and exit through the venous blood outflow. In *Liver* and *RB*, *fT4* and *fT3* are able to move bidirectionally as first-order processes between the tissue blood and tissue proper subcompartments (**Figure 1A**: *k*_21_, *k*_23_, *k*_25_, and k_27_ for influx; *k*_28_, *k*_29_, *k*_30_, and *k*_31_ for efflux). Using first-order processes to describe these exchanges is well justified because although they are membrane transporter-mediated, the binding affinities (*K_m_*) of T4 and T3 for these transporters are normally in the high nM to µM range (30, 31), which are hundreds of times higher than the free T4 and T3 concentrations in the plasma at least (32, 33). The *Thyroid* compartment is simplified by describing zero-order, unidirectional releases of *fT4* and *fT3* into *Thyroid blood* (**Figure 1A**: *k*_20_ and *k*_22_ respectively) while the concentrations of T4 and T3 in *Thyroid tissue per se* are not modeled.

Within the tissue proper of the *Liver* and *RB* compartments, T4 and T3 partition into free and bound forms which are assumed to be in fast equilibrium, such that their free fractions are described by constants (*Fu_T_*_4_ and *Fu_T_*_3_). Only free T4 and free T3 in the tissue proper are metabolized and cleared according to first-order processes. Conversion of T4 into T3 by DIOs is described by the *k*_24_ (*RB*) and *k*_26_ (*Liver*) processes (**Figure 1A**). The metabolism of T4 into other metabolites, including reverse T3, glucuronidated and sulfated T4, which are not explicitly tracked in the model, is described by the *k*_32_ (*RB*) and *k*_34_ (*Liver*) processes. T3 is cleared by the *k*_33_ (*RB*) and *k*_35_ (*Liver*) processes.

After the completion of the model construction, a mass balance check was performed for T4 and T3 by terminating their productions and tracking the amounts of T4 and T3 in various compartments and metabolized over time (**Figure S1**). The sum was then compared with the steady-state amounts before the productions were turned off. As indicated in **Figure S1**, both T4 and T3 in the model are mass balanced.

To investigate the phases of plasma T4 and T3 clearance and their half-lives, TH tracer experiments were simulated, for which T4 tracer or T3 tracer are coded as separate state variables that have identical interactions and properties as the respective endogenous counterparts.

### 2.2 Modeling the effect of EDCs

To examine the effects of THBP-binding EDCs, a chemical *X* is introduced in the PBK model. Reversible binding of *X* to either *TBG* or *TTR* is considered (**Figure 1B**, pink-shaded area). In each blood (sub)compartments, an additional ODE for free *X* and two additional ODEs for the *X*-bound TBG (*XTBG*) or *X*-bound TTR (*XTTR*) complexes are included. For simplicity, *X* is introduced to the model through a zero-order production process in the *Body Blood* compartment, resembling some environmental exposures not explicitly considered here, and *X* is cleared through a first-order process also in the *Body Blood* compartment.

### 2.3 Sensitivity analysis

Local sensitivity analysis was performed by increasing or decreasing the relevant parameters by 1% from the default values and allowing the system to reach steady state. The averaged ratio of the percentage change of T4 or T3 in *Liver* or *RB* to the percentage change in the parameter was calculated as the *sensitivity coefficient*.

### 2.4 Model parameters, equations, and simulation tools

An average human individual of 75 kg body weight is simulated. Model parameter values and details of the source references, justifications, and mathematical calculations from which the parameter values were derived are provided in **Table S1-S3** and their footnotes. The ODEs and algebraic equations are provided in **Table S4**. The models were constructed in MATLAB R2019a (MathWorks Natick, Massachusetts, USA), and *ode15s* was used to numerically solve the ODEs. All MATLAB code is available at https://github.com/pulsatility/2023-TH-PBK-Model.git.

## 3 Results

### 3.1 Basic steady-state characteristics of the PBK model

#### 3.1.1 TH concentrations and distribution in blood

Steady-state TH concentrations and percentage distribution among free and THBP-bound THs in the *Body Blood* compartment are provided in **Table 1**. The plasma concentration of *fT4* is 15 pM, which accounts for 0.016% of *Total T4* in this compartment. *T4TBG* is at 75%, accounting for the majority of *Total T4*, followed by *T4TTR* at 17% and *T4ALB* at 7.8%. The plasma concentration of *fT3* is 5 pM, which accounts for 0.3% of *Total T3* in the *Body Blood* compartment. *T3TBG* is at 75%, accounting for the majority of *Total T3*, followed by *T3ALB* at nearly 20% and *T3TTR* at nearly 5%. The TH concentrations in the *Liver blood* and *RB blood* compartments are slightly lower but nearly indistinguishable from those in *Body Blood* (simulation results not shown). The *fT4* and *fT3* concentrations in the *Thyroid blood* reach 17.4 and 5.9 pM respectively, which are 16 and 18.6% higher than in *Body Blood*, but the THBP-bound THs are only marginally higher.

**Table 1:** Plasma TH concentrations and percentage distribution in *Body Blood* compartment.

	Free TH	TH-TBG	TH-TTR	TH-ALB	Total TH
T4 (pM)	15.0	7.0E4	1.6E4	7.275E3	9.334E4
T4 (% total)	0.0161%	75.04%	17.15%	7.79%	100%
T3 (pM)	5.0	1.276E3	82.2	331.4	1.694E3
T3 (% total)	0.296%	75.3%	4.85%	19.56%	100%

#### 3.1.2 THBP saturation

In the *Body Blood* compartment, TBG is 20.29% saturated, which is contributed predominantly by T4, accounting for 19.93%, while T3 only accounts for the remaining 0.36% (**Table 2**). The values are comparable to the 18.4% or 20% TBG saturation by T4 in the literature (9, 10). TTR is only 0.3% saturated by THs, also consistent with the 0.16% or 0.5% values in the literature (9, 10). TH-bound TTR is also dominated by T4, which accounts for 0.2991%, while T3 only accounts for 0.0015%. ALB is barely saturated by THs, and at 0.0012%, it is comparable to the estimated 0.0016% in the literature (10). TH-bound ALB is dominated by T4, which accounts for 0.0011% and T3 only accounts for 0.00005%. The percentage saturations of THBPs in the *Liver blood* and *RB blood* are slightly lower but nearly identical to those in *Body Blood* (simulation results not shown). The saturations in the *Thyroid blood* are slightly higher than in *Body Blood* because of the binding of THs newly released from the *Thyroid tissue* (**Table 2**).

**Table 2:** Percentage saturation of THBPs by T4 and T3.

		TBG	TTR	ALB
Body Blood	T4	19.93%	0.2991%	0.0011%
	T3	0.36%	0.0015%	5.137E-5%
	T4+T3	20.29%	0.3007%	0.0012%
Thyroid blood	T4	20.07%	0.308%	0.0013%
	T3	0.38%	0.0017%	5.947E-5%
	T4+T3	20.45%	0.31%	0.00132%

#### 3.1.3 TH concentrations and abundance in tissue compartments

The steady-state concentrations and amounts of THs in the tissue compartments are listed in **Table 3**. Total T4 concentrations in the *Liver tissue* and *RB tissue* are 2.26E5 and 9.89E3 pM, respectively. These concentrations are 2.42 and 0.11-fold of plasma total T4 concentration, respectively. This *Liver tissue* to *Plasma* concentration ratio (or partition coefficient) is consistent with the near 3:1 ratio estimated in earlier studies (34). T4 amounts in the entire blood, *Liver tissue*, and *RB tissue* are 2.968E5, 3.878E5, and 6.097E5 pmol, accounting for 23%, 30%, and 47% of all extrathyroidal T4, respectively. The T4 amount in the *Liver tissue* is thus 1.3 times higher than that in the blood, which is consistent with the median value reported in (35). In comparison, total T3 concentrations in the *Liver tissue* and *RB tissue* are 3.51E3 and 790 pM, which are 2.07 and 0.46-fold of total T3 plasma concentration, respectively. T3 in the entire blood, *Liver tissue*, and *RB tissue* are 5.397E3, 6.018E3, and 4.867E4 pmol, accounting for 9%, 10%, and 81% of all extrathyroidal T3, which is consistent with the estimation that over 90% of extrathyroidal T3 is in extravascular compartments (36). The percentages of T4 and T3 amounts in the *Liver tissue*, i.e., 30% and 10% respectively, are comparable to what were reported in the literature (24, 34, 35), as summarized in **Table S2**.

**Table 3:** Concentrations, amounts and percentage distribution of THs in tissue compartments.

	Blood	Liver tissue	RB tissue	Total extrathyroidal tissues
T4 (pM)	9.334E4**	2.261E5	9.893E3	–
T4 (pmol)	2.968E5*	3.878E5	6.097E5	1.294E6
T4 (%)	22.93%	29.96%	47.11%	100%
T3 (pM)	1.694E3**	3.508E3	790	–
T3 (pmol)	5.397E3*	6.018E3	4.867E4	6.007E4
T3 (%)	8.97%	10.02%	81.01%	100%

*It includes amounts in Body Blood, Liver blood, RB blood and Thyroid blood.

**Concentrations in Body Blood.

#### 3.1.4 TH production and metabolism in tissue compartments

The rates of steady-state TH production and metabolism in different tissue compartments are presented in **Table 4**. For an average individual of 75 kg body weight in our model, T4 is produced by the *Thyroid* at 1.6 pmol/S (equivalent to 107.39 µg/day), which is comparable to the values reported (23, 37, 38), as summarized in detail in **Table S1**. At this production rate, the daily turnover of T4 in all extrathyroidal tissues is about 10.68%. Nearly 35% of T4 is eliminated in *Liver* and 65% in *RB*. T3 is produced by the *Thyroid* at 0.1143 pmol/S (6.43 µg/day), accounting for 22% of total T3 production in the whole body, which is close to the 23.7% estimated previously (38). The remaining T3 is produced by T4 conversion in peripheral tissues, where *Liver* contributes 27% and *RB* 51%. This nearly 1:2 ratio is close to the 15:29 ratio estimated for T3 production in liver and muscle in euthyroid condition (39). Therefore, *RB* contributes to nearly 2/3 of T3 produced extrathyroidally. The daily turnover of T3 in all extrathyroidal tissues is about 74%. Peripheral T4-to-T3 conversion accounts for 25% of overall T4 metabolism, among which 8.73% and 16.28% occur in *Liver* and *RB* respectively. The *Liver* metabolizes 40% and *RB* metabolizes 60% of T3 produced overall.

**Table 4:** TH production and metabolism rates.

	Thyroidal Production	Liver		RB	
		Production	Metabolism	Production	Metabolism
T4 (pmol/S)	1.6	na	0.5584	na	1.0416
% of T4 production	100%	na	34.9%	na	65.1%
T3 (pmol/S)	0.1143	0.1396	0.2060	0.2604	0.3083
% of T3 total production*	22.22%	27.15%	40.05%	50.63%	59.95%
% of T4 production	7.14%	8.725%	12.88%	16.28%	19.27%

*T3 total production = 0.1143 + 0.1396 + 0.2604 = 0.5145 pmol/S. na, not applicable.

#### 3.1.5 TH blood supply and blood-tissue exchange

The rates of the steady-state TH blood supply and influx and efflux in the tissue compartments are presented in **Table 5**. For the *Liver* compartment, T4 is supplied through the blood at a rate of 1.33E3 pmol/S for *Total T4* and 0.21 pmol/S for *fT4*. For the exchange of T4 between *Liver blood* and *Liver tissue*, the influx is 63.54 pmol/S and efflux is 62.98 pmol/S, which are much greater than the blood supply rate of *fT4* and only a small fraction of the blood supply rate of *Total T4*. T4 efflux is 99.12% of T4 influx, indicating that only a tiny fraction of T4 coming into the *Liver tissue* is metabolized and the vast majority returns to blood. T3 is supplied to the *Liver* compartment through the blood at a rate of 24.12 pmol/S for *Total T3* and 0.071 pmol/S for *fT3*. T3 enters the *Liver tissue* at a rate of 6.97 pmol/S and exits at 6.91 pmol/S. Similar to the case of T4, these rates are much greater than the blood supply rate of *fT3*. T3 efflux is 99.05% of T3 influx, thus only about 1% (0.0664 pmol/S) coming into the *Liver tissue* is metabolized. The simulation results presented here are consistent with the estimation that >99% of T4 and T3 that enter the liver return to blood unmetabolized (7). T3 is produced from T4 locally in the *Liver tissue* at 0.14 pmol/s, and therefore, local T3 production contributes about 68% of T3 in the *Liver tissue* while uptake from the blood contributes the remaining 32%.

**Table 5:** TH blood supply and exit, blood-tissue flux, tissue production and metabolism.

		Tissue influx	Tissue efflux	Tissue production	Tissue metabolism	Blood supply (total)	Blood exit (total)	Blood supply (free)	Blood exit (free)
Liver	T4 (pmol/S)	63.5368	62.9784	na	0.5584	1329.0136	1328.4552	0.2136	0.2132
	% of T4 influx	100%	99.12%	na	0.88%	2091.7%	2090.8%	0.3362%	0.3356%
	T3 (pmol/S)	6.9731	6.9067	0.1396	0.2060	24.1248	24.0584	0.0713	0.0710
	% of T3 influx	100%	99.05%	2.00%	2.95%	345.97%	345.02%	1.0228%	1.0185%
RB	T4 (pmol/S)	14.9901	13.9486	na	1.0416	3907.2999	3906.2584	0.6280	0.6275
	% of T4 influx	100%	93.05%	na	6.95%	26066%	26059%	4.1894%	4.1861%
	T3 (pmol/S)	0.7507	0.7028	0.2604	0.3083	70.9270	70.8791	0.2097	0.2095
	% of T3 influx	100%	93.62%	34.69%	41.07%	9448.4%	9442%	27.9319%	27.9073%

na, not applicable.

Despite a much larger volume of the *RB* compartment than the *Liver* compartment, T4 influx and efflux in *RB* are much slower, at 14.99 and 13.95 pmol/S respectively, which are about 22%-23% of the corresponding fluxes in the *Liver*. These values are about 22-23 times higher than the *RB* blood supply rate of *fT4*. T4 efflux is 93% of T4 influx, indicating only a small fraction (7%) of T4 coming into the *RB* tissue is metabolized. Similar to the case of T4, T3 influx and efflux in *RB* are much slower that in the *Liver*, at 0.75 and 0.70 pmol/S respectively, which are about 10% of the corresponding fluxes in the *Liver*. The blood-tissue exchange rates are about 3.4 times higher than the blood supply rate of *fT3*. T3 efflux is 94% of the T3 influx, thus 6% (0.048 pmol/S) of T3 coming into *RB* is metabolized. T3 is produced locally at 0.26 pmol/S in *RB*, which is > 5 times higher than T3 transported from the blood into *RB* and is about 35% of the T3 influx rate. The overall metabolism rate of T3 in *RB* is 41% of the T3 influx rate.

The high blood-tissue exchange rates relative to the local production and/or metabolism rates, particularly in the *Liver* compartment, indicate that at the steady state the tissue concentrations of THs are largely determined by the quasi-equilibrium with plasma free THs. This suggests that the steady-state tissue concentrations of T4 and T3 would be relatively insensitive to perturbations of the local metabolism as long as the plasma *fT4* and *fT3* concentrations stay constant, e.g., through the HPT feedback regulation. Sensitivity analysis confirmed such prediction, as all sensitivity coefficients are much lower than unity, particularly in *Liver* (**Table S5**). These results suggest an intrinsic mechanism for local T4 and T3 homeostasis.

### 3.2 Transient TH tissue distribution kinetics and half-lives

#### 3.2.1 Distribution phases and half-lives

**Figure 2:**
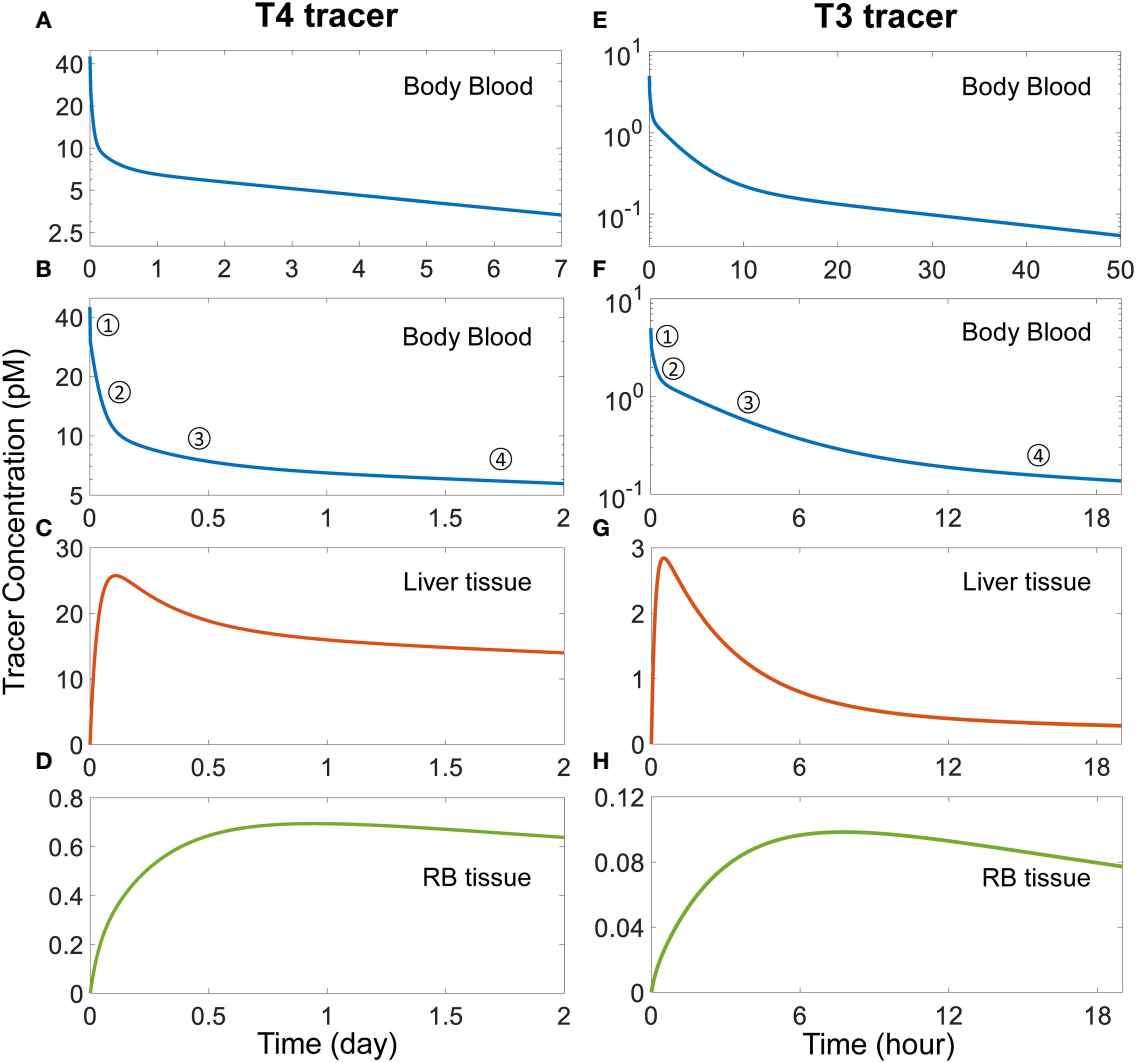
Simulation of TH tracer experiments to determine distribution phases and apparent half-lives of T4 and T3 in the PBK model. For the T4 tracer simulation, 45 pM of *fT*4 tracer is added to the *Body Blood* compartment at time 0. **(A)** Plasma concentration of total T4 tracer in *Body Blood* over time. **(B)** Magnified view of **(A)** to highlight the four T4 phases as indicated. **(C-D)** T4 tracer concentrations in *Liver tissue* and *RB tissue* respectively. For the T3 tracer simulation, 5 pM of *fT*3 tracer is added to the *Body Blood* compartment at time 0. **(E)** Plasma concentration of total T3 tracer in *Body Blood* over time. **(F)** Magnified view of **(E)** to highlight the four T3 phases as indicated. **(G-H)** T3 tracer concentrations in *Liver tissue* and *RB tissue* compartments respectively.

With the basic steady-state characteristics of the model largely in line with the literature, we next simulated TH tracer experiments to examine the phases of plasma and tissue T4 and T3 transients and their half-lives so derived. With a T4 tracer 3 times the concentration of *fT4* introduced to the *Body Blood* compartment (45 pM), T4 tracer is cleared in 4 distinct phases (**Figures 2A, B**). The 1^st^ phase is the rapid distribution of the T4 tracer from the *Body Blood* to the *Liver blood* and *RB blood* compartments, which is completed in less than a minute. This step dilutes plasma T4 tracer in the *Body Blood* from 45 pM to 30 pM by the approximate 2:1 plasma volume ratio between *Body Blood* and *Liver blood*+*RB blood*. The 2^nd^ phase is the distribution to the *Liver tissue* compartment, as evidenced by the peaking of T4 tracer in *Liver tissue* at about 2.6 h or 0.11 day (**Figure 2C**). The 3^rd^ phase is the distribution to the *RB tissue* compartment, as evidenced by the peaking of the T4 tracer in *RB tissue* at 22.66 h or 0.94 day (**Figure 2D**). Lastly, the 4^th^ phase is the metabolism phase. The half-life of plasma T4 tracer, calculated based on the slope of the 4^th^ phase, is about 6.43 days.

For the simulated T3 tracer experiment, T3 tracer is also cleared in 4 distinct phases (**Figures 2E, F**), with the 1^st^ phase being the distribution between blood compartments, the 2^nd^ phase the distribution to *Liver tissue*, completed at 0.5 h (**Figure 2G**), the 3^rd^ phase the distribution to *RB tissue*, completed at 7.8 h (**Figure 2H**), and the 4^th^ phase the metabolism phase. The half-life of plasma T3 tracer, calculated based on the slope of the 4^th^ phase, is about 23.56 hours.

#### 3.2.2 Effects of blood flow rate

Several factors can interplay to determine how fast T4 and T3 are taken up by tissues, including the blood flow rate, the transporter-mediated exchange rates between the tissue proper and tissue blood, and the availability of plasma THs owing to THBP binding. Among these factors, we first explored the blood flow rate by varying the cardiac output QC. Increasing QC accelerates the 1^st^ phase, i.e., the distribution of T4 tracer from *Body Blood* (**Figure S2A**) into *Liver blood* (**Figure S2B**) and *RB blood* (**Figure S2C**), while the opposite occurs when decreasing QC. While the blood flow rate affects the duration of the 1^st^ phase of plasma T4 tracer clearance, the remaining 3 phases are largely unaffected (**Figures S2D–F**). Similar results were obtained for T3 tracer simulations with varying QC (**Figure S3**).

#### 3.2.3 Effects of blood-tissue exchange rates

We next explored the exchange rates between the blood and tissues. When both *k*_25_ and *k*_30_, the two parameters governing T4 exchange in *Liver*, are doubled simultaneously, which leaves the basal T4 level in *Liver tissue* largely unchanged, the T4 tracer peak in *Liver tissue* becomes higher and advanced from 2.6 h (0.11 day) to 1.73 h (0.072 day) (**Figure S4B**). When *k*_25_ and *k*_30_ are halved simultaneously, the T4 tracer peak in *Liver tissue* becomes lower and delayed to 4.07 h (0.17 day). The peak time in *RB tissue* is unaffected except that the rising phase becomes either slightly slower or faster (**Figure S4C**), which results from the altered T4 tracer distribution into *Liver tissue*. Therefore, the T4 influx and efflux rate constants in *Liver* play an important role in determining how fast T4 is taken up by the *Liver tissue*, which in turn determines the duration of the 2^nd^ phase of T4 tracer clearance in *Body Blood* (**Figure S4A**).

Similarly, when *k*_21_ and *k*_28_ for T4 influx and efflux in *RB* are doubled simultaneously, which also leaves the basal T4 level in *RB tissue* largely unchanged, the T4 tracer peak in *RB tissue* becomes higher and advanced from 22.65 h (0.94 day) to 13.68 h (0.57 day) (**Figure S4F**). When *k*_21_ and *k*_28_ are halved simultaneously, the T4 tracer peak in *RB tissue* becomes lower and delayed to 36 h (1.5 day). As a consequence, the T4 peak in *Liver tissue* is also affected to some extent, which becomes lower but advanced to 2.22 h (0.092 day) or higher but delayed to 3.04 h (0.13 day) as *k*_21_ and *k*_28_ are doubled or halved, respectively (**Figure S4E**). Therefore, the T4 influx and efflux rate constants in *RB* play an important role in determining how fast T4 is distributed into the *RB tissue*, which in turn determines mainly the duration of the 3^nd^ phase of T4 tracer clearance in *Body Blood* (**Figure S4D**).

While affecting the T4 distribution phases, varying neither *k*_25_ and *k*_30_ nor *k*_21_ and *k*_28_ has a tangibly impact on the half-life of T4. Similar effects on the timing and duration of the 2^nd^ and 3^rd^ phases of plasma T3 clearance can be also observed for T3 tracer simulations when the blood-tissue exchange rate constants, *k*_27_ and *k*_31_ for *Liver* and *k*_23_ and *k*_29_ for *RB*, are varied respectively (**Figure S5**). Taken together, these results suggest that how fast the membrane transporters can move THs in and out of tissues play a key role in transient TH distribution kinetics.

#### 3.2.4 Effects of THBP availability

**Figure 3:**
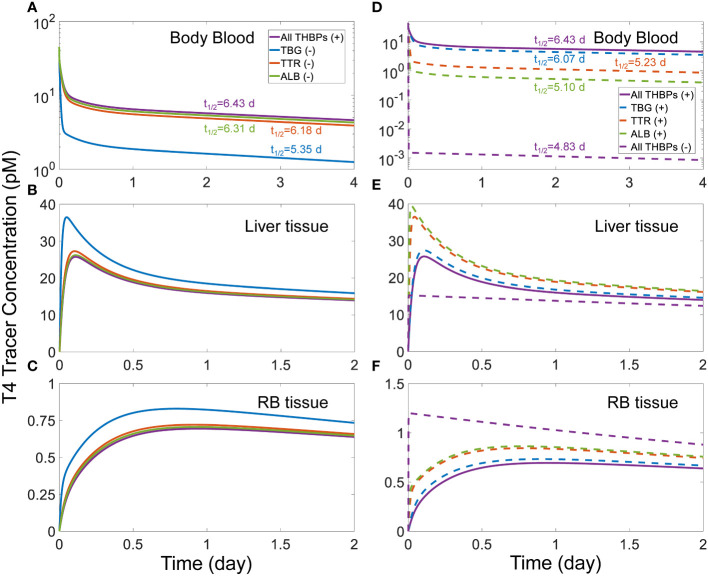
Effects of THBP availability on T4 distribution kinetics and half-life. For the T4 tracer simulation, 45 pM of *fT*4 tracer is added to the *Body Blood* compartment at time 0. Concentrations of T4 tracer in *Body Blood* **(A)**, *Liver tissue* **(B)**, and *RB tissue* **(C)** over time with only one THBP removed as indicated in **(A)**. Concentrations of T4 tracer in *Body Blood* **(D)**, *Liver tissue* **(E)**, and *RB tissue* **(F)** over time with two or all three THBPs removed as indicated in **(D)**. T4 half-life is indicated for each condition.

We next explored how the availability of THBPs can affect TH clearance and half-lives. As TBG, TTR, and ALB are sequentially removed from the system, the 2^nd^ phase and, to a lesser extent, the 3^rd^ phases of T4 tracer clearance are progressively advanced and the half-life becomes shorter (**Figure 3**). When only TBG is removed (solid blue line), the *Liver* peak of T4 tracer (**Figure 3B**) is advanced from 2.6 h (0.11 day) to 1.13 h (0.047 day) and reaches a higher level, the *RB* peak (**Figure 3C**) is advanced from 22.66 h (0.94 day) to 18.96 h (0.79 day) also reaching a higher level, and the T4 half-life is shortened from 6.43 day to 5.35 day (**Figure 3A**). Compared with TBG, removing TTR or ALB has a similar but much minor effect (**Figures 3A–C**, solid orange and green lines respectively). As any two of the three THBPs are removed from the system, similar results occur, with the removal of TBG+TTR combination producing the largest effect (**Figures 3D–F**, dashed green line). Therefore, the effect size is positively correlated to the total abundance of T4-THBPs removed. The advancement of the *Liver* and *RB* distribution phases of blood T4 tracer clearance occurs because as THBPs are removed, fewer T4 tracer molecules are bound and more free T4 tracer becomes available in the blood; as a result, T4 tracer translocates into *Liver tissue* and *RB tissue* faster, reaching a higher peak and equilibrating with blood sooner. The shortening of the T4 half-life is because as the *Total T4* in the blood is reduced with some THBPs removed, the relative abundance of T4 in the tissues becomes higher; since T4 is metabolized only in the tissues not in the blood, the overall half-life becomes shorter. In the extreme case when all THBPs are absent, the quasi-equilibrium between blood and tissues is achieved extremely fast, in only a few minutes (**Figures 3D–F**, dashed purple line). This suggests that the process of free T4 transportation into the tissues *per se* is not limiting and it becomes limiting only when plasma T4 is considerably stored in THBPs. In the absence of all three THBPs, the half-life of plasma T4 tracer decreases to 4.83 days.

**Figure 4:**
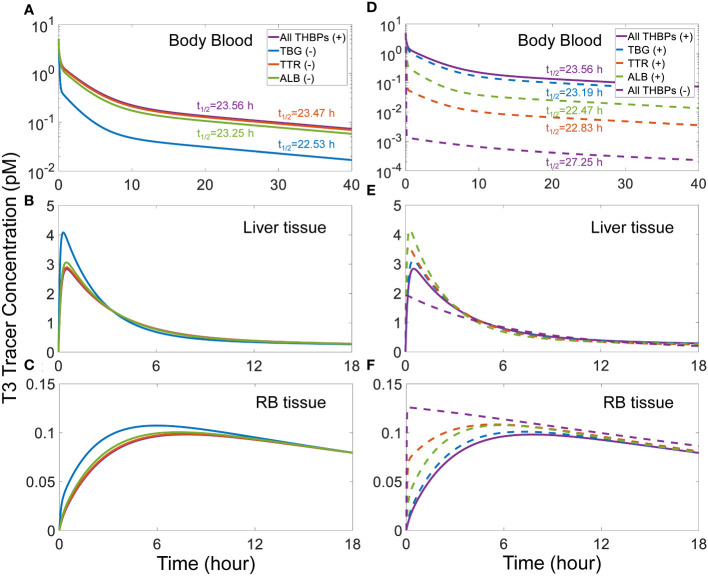
Effects of THBP availability on T3 distribution kinetics and half-life. For the T3 tracer simulation, 5 pM of *fT*3 tracer is added to the *Body Blood* compartment at time 0. Concentrations of T3 tracer in *Body Blood* **(A)**, *Liver tissue* **(B)**, and *RB tissue* **(C)** over time with only one THBP removed as indicated in **(A)**. Concentrations of T3 tracer in *Body Blood* **(D)**, *Liver tissue* **(E)**, and *RB tissue* **(F)** over time with two or all three THBPs removed as indicated in **(D)**. T3 half-life is indicated for each condition.

A similar trend of phase advancement and slightly shortened half-life also occurs for T3 tracer as each THBP species is removed from the system (**Figure 4**), with the counterintuitive exception that in the absence of all three THBPs, the T3 half-life is lengthened to about 27 hours. This can be explained by the vastly different rate constants of T3 metabolism in the *RB tissue* vs. *Liver tissue* as detailed in **Figure S6**.

#### 3.2.5 Effects of TH-THBP binding rates

Given the effects of THBP availability in the transient TH distribution kinetics as revealed above, we next explored the effects of how fast THs can be released by THBPs. This is done by varying the pair of association and dissociation rate constants of TH-THBP binding simultaneously by the same fold, which would leave the binding affinities unchanged. When the pairs of *k*_1_ and *k*_2_, *k*_3_ and *k*_4_, and *k*_5_ and *k*_6_ are all increased by 10-fold simultaneously, the T4 tracer *Liver* peak is slightly advanced from 2.6 h (0.11 day) to 2.4 h (0.1 day), while a 10-fold decrease delays the peak to 3.49 h (0.15 day) and also lowers the peak level (**Figure S8B**). Correspondingly the 2^nd^ phase of the plasma T4 tracer clearance is also advanced or delayed respectively (**Figure S8A**). These parameter variations do not affect the T4 trace *RB* peak (**Figure S8C**). These results suggest that how fast T4 can be released by THBPs also play a role, albeit minor, in T4 *Liver* distribution kinetics. For the T3 tracer simulation, varying both the association and dissociation rate constants for T3 binding to THBPs, including the pairs of *k*_7_ and *k*_8_, *k*_9_ and *k*_10_, and *k*_11_ and *k*_12_, simultaneously by the same fold, has a similar but even smaller effect than the case of T4 (**Figures S8D–S8F**).

### 3.3 Effects of THBP-binding EDCs

A major concern with EDCs targeting the thyroid system is that some of them are structurally similar to the two-ringed T4 and T3 molecules and may compete with THs for target proteins including the THBPs. If an EDC is able to avidly bind to a THBP species, the THs bound to that THBP may be displaced and liberated into the plasma, causing an increase in free THs, at least transiently. To explore these situations, we assume a hypothetical reference compound *X* that has the same binding affinities and rate constants as T4 for TBG or TTR.

**Figure 5:**
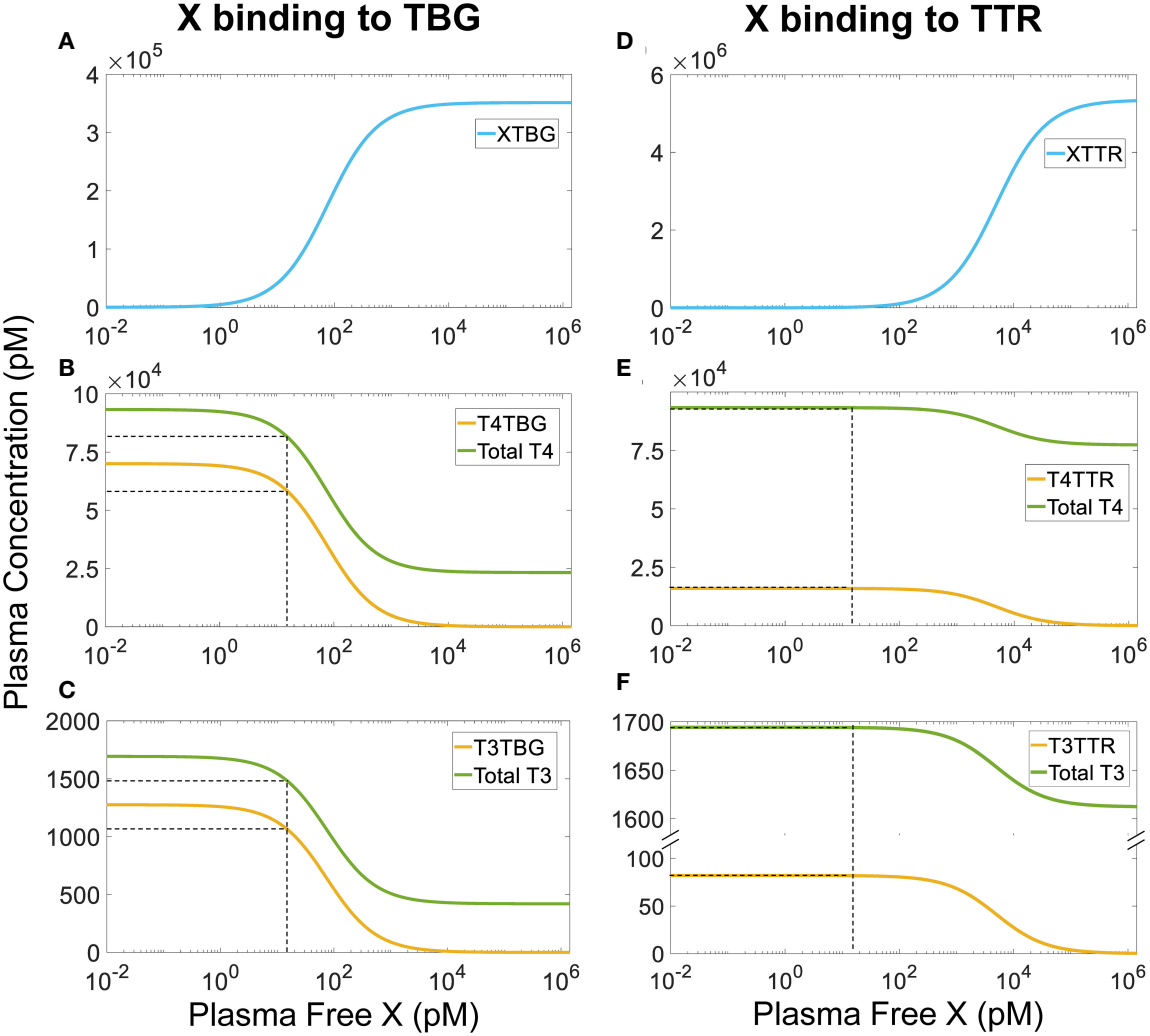
Steady-state plasma concentrations of TH-THBPs and X-THBPs when an EDC *X* competes for binding to either TBG **(A-C)** or TTR **(D-F)** with the same binding affinities and rate constants as T4. Concentration-response curves for *XTBG* **(A)**, *T4TBG* and *Total T4* **(B)**, *T3TBG* and *Total T3* **(C)**. Concentration-response curves for *XTTR* **(D)**, *T4TTR* and *Total T4* **(E)**, *T3TTR* and *Total T3* **(F)**. Dashed lines indicate effects when *free X* concentration equals *fT4* concentration (15 nM).

Our simulations demonstrated that for continuous, long-term exposure where plasma *X* reaches a steady state, its effect on the steady-state *fT4* and *fT3* levels is negligible regardless of the *X* concentrations (simulation results not shown). When *X* is able to bind to TBG, the steady-state plasma *XTBG* level increases in a typical Michaelian manner as *free X* increases (**Figure 5A**). As a reference point, at a *free X* concentration that is the same as basal *fT4*, i.e., 15 pM, *T4TBG* decreases by 16.57% (**Figure 5B**, yellow line) while *Total T4* decreases by 12.4% (**Figure 5B**, green line). *T3TBG* decreases by 16.93% (**Figure 5C**, yellow line) while *Total T3* decreases by 12.63% (**Figure 5C**, green line). For *T4TBG* and *Total T4* to decrease by 10% from their basal values, the required concentration of *free X* is 8.5 and 11.56 pM respectively. For *T4TBG* and *Total T4* to decrease to 50% of their basal values, the concentration of *free X* must increase to 75 and 150 pM respectively. Despite these wide changes in *T4TBG* and *Total T4*, *fT4* barely changes, and neither do *T4TTR*, *T4ALB*, and T4 in *Liver tissue* and *RB tissue* (simulation results not shown). The *free X* concentrations causing 10% and 50% drops in *T3TBG* and *Total T3* are similar to the case of T4. *fT3*, *T3TTR*, *T3ALB*, and T3 in *Liver tissue* and *RB tissue* only have negligible changes (simulation results not shown).

A similar but much minor effect is observed when *X* binds to TTR only (**Figures 5D–F**). At 15 pM, *X* only causes steady-state *T4TTR* and *Total T4* to decrease by a tiny 0.001%, and *T3TTR* and *Total T3* by 0.0001%. The lessor effect is because the reserve capacity of TTR (percentage TTR unoccupied by THs) is much higher (about 99.7%) than that of TBG (about 80%) (**Table 2**). Therefore, at the same *free X* concentration, fewer TH molecules will need to be displaced from TTR than from TBG since there are so many more unoccupied TTR molecules available for *X*. For *T4TTR* to decrease to 90% and 50% of its basal value, the concentration of *free X* must increase to 550 and 5000 pM, respectively. *T3TTR* has basically the same percentage drops at these two *X* concentrations. Unlike the case when *X* can bind to TBG, here *total T4* and *total T3* never decrease to levels lower than 82.5% and 95% of their basal values, respectively. *fT4*, *fT3*, other bound forms, and tissue THs only have negligible changes (simulation results not shown). Taken together, continuous exposure to THBP-binding EDCs does not perturb the free TH homeostasis.

**Figure 6:**
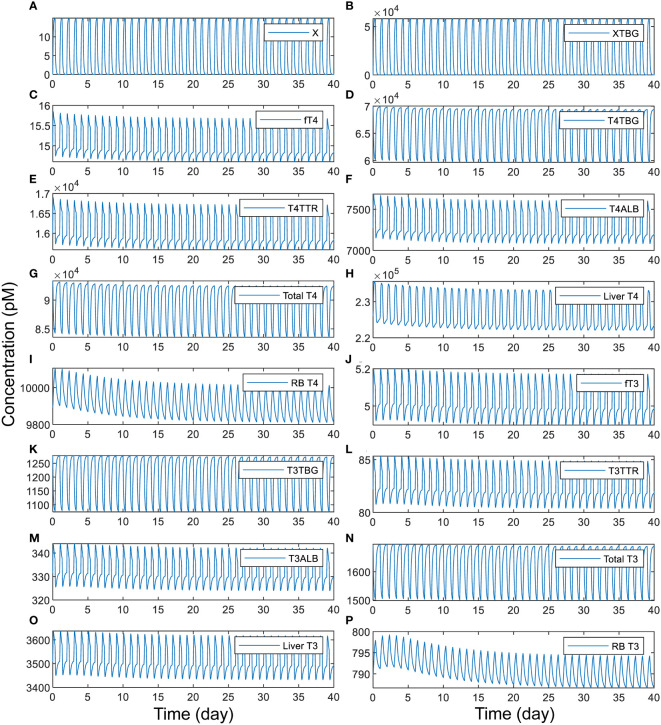
Time-course responses of X and TH species as indicated (A-P) to 8-h daily exposure to TBG-binding *X* that has a half-life of 1 h. The simulation of exposure to *X* begins from the basal steady state of the PBK model. The exposure level is set such that if the exposure is continuous for 24 h each day, it produces a steady-state plasma *free X* concentration of 15 pM. *X* binds to TBG with the same affinity and rate constants as T4.

We next explored the situation of intermittent, daily exposure, where *X* can be quickly metabolized with a short half-life such that its plasma concentration fluctuates throughout the day. Our simulations demonstrate that when *X* has an overall half-life of 1 h, a daily 8-h exposure to TBG-binding *X* can cause large spikes of plasma *free X* (**Figure 6A**) and *XTBG* (**Figure 6B**), which are cleared nearly completely at the end of the day. When the peak level of *free X* reaches 15 pM, the perturbation is sufficient to cause 7% daily fluctuation of *fT4* (**Figure 6C**) and 5.4% of *fT3* (**Figure 6J**), 4.8% of T4 (**Figure 6H**) and 5.3% of T3 (**Figure 6O**) in *Liver tissue*, and 2% of T4 (**Figure 6I**) and 1% of T3 (**Figure 6P**) in *RB tissue*. T4-THBPs and T3-THBPs all exhibit daily fluctuations of various degrees (**Figures 6D–F, G, K–N**).

**Figure 7:**
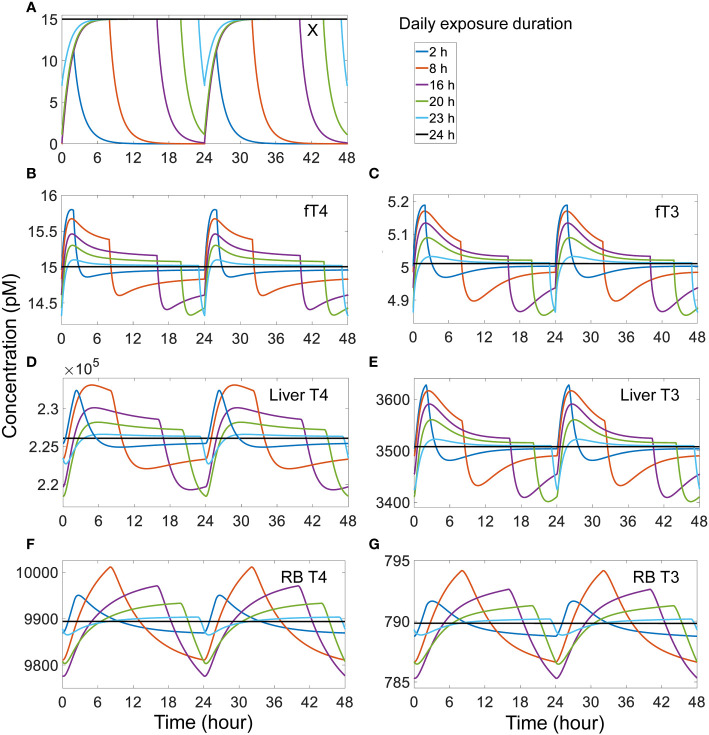
Effects of different duration of daily exposure to TBG-binding *X* that has a half-life of 1 h on X and TH species as indicated (A-G). The model was run from the basal steady state for 52 days of exposure such that a steady-state range of fluctuation was achieved. The responses on day 51-52 (labeled as 0-48 h) are shown. The exposure level is set such that if the exposure is continuous for 24 h each day, it would produce a steady-state plasma *free X* concentration of 15 pM. *X* binds to TBG with the same affinity and rate constants as T4.

When the daily exposure duration becomes shorter, *fT4*, *fT3*, and tissue T4 and T3 tend to spike up to higher peak levels and dip to higher trough levels, whereas when the daily exposure duration becomes longer, the opposite occurs (**Figure 7**). These changes are at first unexpected because with a longer exposure, more THs are expected to be displaced from their THBP-bound forms and thus free THs are expected to rise to higher levels. This counterintuitive phenomenon can be explained as follows. Because of the fast binding kinetics, the T4 and T3 displaced from TBG due to competitive *X* binding is initially absorbed by TTR and ALB and a quasi-steady state is quickly established in minutes, like in a tracer experiment. With a longer daily exposure duration, the displaced plasma T4 and T3 will have more time to be distributed from the blood to tissues and metabolized therein, allowing plasma *fT4* and *fT3* to settle back closer to their basal steady-state levels before the exposure is terminated for the day (**Figures 7B, C**). At the moment the exposure is terminated, *X* drops quickly (**Figure 7A**) and those TBG bound by *X* is thus released. These “new” free TBG molecules will mop up plasma *fT4* and *fT3*, causing them to undershoot below the steady-state levels (**Figures 7B, C**). Since with a longer daily exposure duration plasma *fT4* and *fT3* are already settling at lower levels during the exposure, as reasoned above, the termination of exposure will cause *fT4* and *fT3* to drop to even lower levels. During the no-exposure period, *fT4* and *fT3* recover some but less so with no-exposure period being shorter. The above pattern repeats as the next-day exposure starts again. The tissue T4 and T3 changes lag behind *fT4* and *fT3* except for *Liver* T3, and they reach higher peak and trough levels with shorter exposure duration (**Figures 7D–G**). Exceptions occur when the exposure duration is either too short or too long. When the duration is too short (2 h or less, **Figure 7** blue lines), the extra plasma T4 and T3 displaced by *X* do not have as much time to move into the tissues, thus the tissue peak levels tend to be lower. When the exposure duration is too long (>23 h, **Figure 7** cyan lines), there is not enough time for tissue T4 and T3 to move back to the blood when the plasma *fT4* and *fT3* dip, resulting in a smaller undershoot in the tissues. With 24-h continuous exposure, all hormone species reach steady states without fluctuations (**Figure 7** black lines).

Increasing the half-life and thus the stability of *X* while keeping the intermittent, daily exposure duration the same decreases the amplitude of hormone fluctuations. With a half-life of 10 h, the exposure only yields steady-state fluctuations of 1.3% in *fT4* and 1.0% in *fT3*, 1.3% and 1.1% in *Liver* T4 and T3 respectively, and 0.5% and 0.3% in *RB* T4 and T3 respectively (**Figure S9**). With a half-life of 100 h, the steady-state fluctuations decrease even further, producing changes of less than 0.1% for all TH species (**Figure S10**). Unlike the case of TBG, 8-hour intermittent, daily exposure to *X* that binds to TTR only produces negligible fluctuations even with a half-life of 1 h (**Figure S11**). The TTR-binding *X* induces daily fluctuations of around 0.03% in *fT4* and 0.006% in *fT3*, 0.019% and 0.006% in *Liver* T4 and T3 respectively, and 0.008% and 0.0009% in *RB* T4 and T3 respectively. For the longer *X* half-lives of 10 and 100 h, the fluctuations are even smaller (**Figures S12-S13**).

## 4 Discussion

In the present study, we built a minimal PBK model that quantitatively recapitulates the production, distribution, and the metabolism of THs for an average human individual. The model is rigorously defined by utilizing and estimating parameters and measurements reported in a multitude of studies in the literature as detailed in **Tables S1-S3** and their footnotes, including THBP binding affinities, association/dissociation rate constants, TH and THBP concentrations, plasma/tissue partitioning, and half-lives, etc. The model allows for the exploration of many features of TH kinetics and provides several novel biological insights into the homeostatic roles of THBPs and the impact of EDCs.

### 4.1 Partitioning of plasma THs among THBPs

For plasma T4, *T4TBG* is the predominant form (75%) followed by *T4TTR* and then *T4ALB*. For T3, *T3TBG* is still the most abundant (75%), but *T3ALB* is more abundant than *T3TTR*. The percentage distributions of plasma TH abundance captured by our model (**Table 1**) are consistent with the ranges reported in the literature as detailed in **Table S2**. The relative distributions of THs among these THBPs are determined by both the respective binding affinities of THs for and the abundances of THBPs. ALB is the most abundant THBP with a molar concentration > 100-fold higher than TTR which in turn is > 10-fold higher than TBG (9, 40). However, the binding affinities of T4 for these THBPs follow a reverse order with drastically different values (8, 9, 41–43), which results in an order of *T4TBG*>*T4TTR*>*T4ALB* for their abundance. Although the binding affinities of T3 for these THBPs also follow an order that is the converse to their abundances, the gap between the binding affinities of T3 for TTR and ALB is not large enough to compensate for the abundance difference between the two THBPs, resulting in a *T3ALB* concentration that is 4-fold higher than *T3TTR*. As a homotetramer, TTR has two binding sites for THs; however, there exists negative cooperativity. This results in a 100-fold difference in the binding affinity for the two sites (44), which justifies the use of a single site for the binding as done in our model here. Absent this negative cooperativity, the *T3TTR* abundance may double, but will still be lower than *T3ALB*.

In the present model, *fT4* is 0.016% of plasma *Total T4*, which is slightly more than half of the 0.03% value often cited in the literature (7–9). 0.03% could be an overestimate of the free T4 fraction for a number of reasons. In our model, to increase *fT4* fraction by nearly 2-fold, i.e., from 0.016% to 0.03%, it will require either (1) reducing the total THBP abundance by nearly half, (2) reducing the binding affinities of T4 for THBPs by nearly half such that plasma *Total T4* will be halved, (3) doubling *fT4* and reducing the binding affinities of T4 for THBPs by nearly half, or some combinations of these. Adjusting only TBG-related parameters may achieve such a result because of its dominance. All these manipulations will require dramatic departure of many parameters or hormone levels from the reference values. Moreover, measuring free T4 using the traditional immunoassays tends to overestimate its concentrations compared with using modern liquid chromatography with tandem mass spectrometry-based reference method (45). It has been reported by laboratories that free T4 fraction can range between 0.005-0.03% (46). Schussler provided an estimate of 0.02% (10), and the free T4 fraction calculated from the mean free T4 and total T4 in the thyroid profile data of the National Health and Nutrition Examination Survey (NHANES) 2007-2012 cycles is slightly over 0.01% (33). Therefore, 0.03% likely represents an upper bound of the free T4 fraction, and 0.016% in our model may be closer to the average value. In contrast, *fT3* in the present model is 0.3% of plasma total T3, which is consistent with the literature 0.3%-0.4% values (7–9).

### 4.2 Influx, efflux, and tissue TH homeostasis

Although THs are highly lipophilic, they do not diffuse through cell membrane readily (31, 47). Earlier studies demonstrated that the transportation of THs across the cell membrane is saturable, indicating a transporter-mediated process (48, 49). Membrane transporters such as monocarboxylate transporters MCT8/10 and those in the organic anion transporter polypeptides (OATP) family are required to move THs across the cell membrane (31, 47). For both T4 and T3 in both the *Liver* and *RB*, the paired influx and efflux rates are comparable and high, and the difference, which is equal to the net influx rate of T4 or T3, is only a tiny fraction of either of the two rates. For the *Liver* compartment, our model is consistent with the estimation that >99% of T4 and T3 that enter the liver return to blood unmetabolized (7). With the flux rates much higher than the tissue metabolism rates, the free THs in the tissue are near equilibrium with the plasma free THs. This suggests that as long as the HPT feedback regulation is able to maintain plasma free TH concentrations relatively constant, alterations in the TH metabolism in the tissue would have minor or even negligible impact on their concentrations in the tissue, especially for T4 in the liver, as demonstrated with the sensitivity analysis (**Table S5**). In contrast, if the metabolism rate is a significant fraction of the unidirectional influx rate, then the tissue concentrations of THs will change markedly with the metabolism constants even though the plasma concentrations can remain unchanged. Therefore, this equilibrium mode of operation may provide some intrinsic robustness against intra-tissue perturbations. This is in addition to other known local feedback and feedforward mechanisms mediated by DIOs that regulate tissue T3 homeostasis (50–54).

### 4.3 TH tracer kinetics and half-lives

#### 4.3.1 TH tissue distribution phases

The T4 tracer simulation showed that the first 3 distribution phases are completed in about one day, which is consistent with experimental observations (23, 55). The 2^nd^ phase reflects the distribution to the *Liver tissue* compartment, as evidenced by the peaking of T4 tracer in *Liver tissue* at about 2.6 h, which is similar to the observations in T4 tracer experiments (34). Likewise, the 3^rd^ phase reflects the distribution to the *RB tissue* and is completed in almost a day which is on par with experimental observations (23). T3 tracer simulation exhibits a similar but faster kinetic profile which is also consistent with experimental observations (23, 38).

Several factors may interplay to determine how fast a T4 or T3 tracer moves into a tissue, including blood flow rate, tissue influx and efflux rates, plasma free TH fractions, and THBP binding kinetics. By themselves, the influx and efflux of THs between blood and tissues *per se* are not limiting. This is because in the absence of THBPs, the tracer in the blood can reach equilibrium with tissues in a few minutes (**Figures 3**, **4**), far quicker than the hours it takes when THBPs are present. Examining the parameter values of the rate constants governing the influx and efflux reveals that they are high enough such that the blood-tissue equilibrium can be reached in seconds or less in the absence of other limiting factors. Without THBPs, the blood mixing or circulation rate is the limiting factor.

Interestingly enough, the influx and efflux become a limiting factor for TH tracer tissue distribution when THBPs are present. This is demonstrated by the high sensitivity of the *Liver* peak and *RB* peak time of TH tracers to changes of the influx and efflux rate constants (**Figures S4**, **S5**). When THBPs are present, because the binding kinetics is much faster than the influx/efflux kinetics, the vast majority of the tracer is first bound by THBPs. With plasma free tracer at very low level, it takes time for the tracer to be released by THBPs and move into the tissues. Increasing the influx and efflux rates will accelerate this process by clearing plasma free tracer more quickly, which will shift the association and dissociation balance of tracer-THBPs to favor unloading more tracer. Consistently, when fewer THBPs are available, less tracer will be bound, resulting in higher plasma free tracer level, which will accelerate the distribution into the tissues (**Figures 3**, **4**). In a similar manner, because the free fraction of plasma T3 is almost 20 times higher than that of T4, the *Liver* peak of T3 tracer occurs much earlier than the *Liver* peak of T4 tracer. As far as the effect of the speed of tracer unloading by THBPs is concerned, increasing the binding rate constants from the default values does not advance the *Liver* and *RB* peaks of the tracer, indicating that the unloading is not rate-limiting in physiological conditions. However, decreasing the binding rate constants by 10-fold delays the *Liver* peak without affecting the *RB* peak (**Figure S8**), suggesting the speed of unloading becomes limiting only under severely altered conditions.

Taken together, the tissue influx and efflux rates are the most limiting steps in the TH tracer distribution kinetics. This is further supported by the following facts. The T4 influx rate constants *k*_25_ = 4.243 L/S for *Liver* and *k*_21_ = 1 L/S for *RB*. At *Liver* and *RB* blood flow rates of 0.0142 L/S and 0.0419 L/S respectively, the equivalent total T4 blood supply rate constants are 88 L/S and 260 L/S respectively. The influx:blood supply ratio is thus 0.048 and 0.0038 for *Liver* and *RB* respectively, which is much lower than unity. This indicates that T4 tissue uptake is a transporter-limited, not a blood flow-limited process, consistent with the conclusion above. Similarly, the influx:blood supply ratio for T3 is 0.29 and 0.011 for *Liver* and *RB* respectively, also consistent with the conclusion that T3 tissue uptake is transporter-limited.

#### 4.3.2 TH half-lives

The half-life of T4, calculated based on the slope of the 4^th^ decay phase in the *Body Blood* compartment, is 6.43 days, which is close to the 6.5 days as estimated using the T4 production rate and the total amount of T4 in the extrathyroidal whole body in the present model. In comparison, the simulated T3 tracer experiment predicts T3 half-life to be 23.6 hours, which is slightly longer than the expected 22.5 hours. While the difference is small, it is worth some further investigation. Examining the T3 tracer amounts in *Liver tissue* and *RB tissue* during the 4^th^, metabolism phase revealed that *RB* contains about 10 times more T3 tracer than *Liver* does (**Figure S7**), while at the basal steady state the *RB : Liver* ratio for the endogenous T3 amount is about 8:1 (**Table 3**). Since the T3 metabolism rate constant in *RB*, *k*_33_, is at least 5 times smaller than that in *Liver*, *k*_35_, a higher T3 tracer distribution compared to the endogenous T3 in *RB* indicates that the overall T3 tracer metabolism in the whole body would be slower than the endogenous T3, leading to a longer half-life of T3 tracer. The reason T3 tracer has a higher *RB : Liver* ratio than the endogenous T3 is due to the following. Assume the *RB : Liver* ratio of T3 tracer amount is 8:1 at some time point, i.e., the same as the endogenous T3. Because T3 tracer is eliminated in *Liver* with a faster rate constant than in *RB*, over time T3 tracer in *Liver* will drop faster than in *RB* percentage-wise. The initial 8:1 ratio is thus not sustainable without continuous supply of T3 tracer, unlike the endogenous T3 which is constantly produced locally to compensate for the faster loss in *Liver*. As a result, the *RB : Liver* ratio of the T3 tracer will decrease continuously until a higher ratio value is reached such that T3 tracer amounts in *RB* and *Liver* decline at the same rate percentage-wise (**Figure S7**). The reason that the T4 half-life predicted by the simulated T4 tracer experiment is largely in line with expectation is because the T4 metabolism rate constants in *RB* and *Liver* are comparable. In summary, the result suggests that clinically measured T3 half-life using radioisotope may be slightly overestimated.

As expected, by holding a significant fraction of THs in the blood where they are not metabolized, the presence of THBPs lengthens the half-lives of THs, which is demonstrated by the sequential removal of THBPs from the system. The only exception is when all THBPs are absent, the T3 half-life is lengthened considerably from 23.5 h to 27 h. Similar to the mechanism above, this lengthening occurs because of the drastically different metabolism rate constants in *RB tissue* vs. *Liver tissue*, which leads to a more predominant distribution of T3 tracer into *RB tissue* (**Figure S6**).

The half-lives of T3 and T4 are altered under pathological thyroid conditions. In general, the half-lives decrease in hyperthyroid patients, and increase in hypothyroid patients (37, 55, 56). As mentioned above, the local feedback and feedforward regulation of T4 and T3 metabolism in the tissues may be the main contributors to the altered half-lives of THs under hyper/hypothyroid conditions. In addition, hyperthyroid patients are expected to have higher TBG saturation, therefore the total T4 in the blood would not increase linearly as more T4 is produced by the thyroid gland. More T4 will tend to be fractioned into the tissues where it is metabolized, contributing to shortened half-lives. Insufficiency or lack of THBPs generally also leads to shorter half-lives of THs because of the shift of THs distribution to tissues as discussed above. Indeed, markedly shortened T4 half-lives have been observed clinically in individuals with TBG deficiency (34).

### 4.4 Effects of EDCs

Many EDCs are structurally similar to THs and can perturb various aspects of the thyroid system (20). Some of them are capable of competing with THs for binding to THBPs such as TTR and TBG with comparable or even higher binding affinities, including PCBs, PBDEs, PBPs, TBBPAs, dibenzo-p-dioxins, and dibenzofurans (13–18). The testing for THBP binding was usually done with *in vitro* T4 displacement assays, which often use high, saturating concentrations of T4 (15, 18). Under such condition, nearly no free TBG or TTR is available to absorb the EDC added to the assay, and T4 already bound to TBG or TTR will be displaced. While *in vivo*, none of the THBPs are saturated more than 25%, and hence there are plenty unbound THBPs that can absorb the EDCs, and the displaced THs from one THBP species can also be quickly absorbed by the other two THBP species. Therefore, results from *in vitro* competitive binding assays with saturating T4 ligand may overestimate the impact and health risk *in vivo* and caution should be exercised for regulatory decision-making when using *in vitro* to *in vivo* extrapolation in such settings.

As demonstrated in our simulations, in the situation of constant exposure, for an EDC that has the same binding affinity for TBG as T4, the plasma total T4 and T3 can drop by about 12.5% when the EDC reaches the same plasma concentration as free T4. In the case of TTR-binding EDCs, the total T4 and T3 drops are negligible. The declines of total THs are not associated with changes in steady-state free T4 and free T3 levels as they are determined by the HPT axis. In a recent study by Hamer et al., housing dust mixtures containing per- and polyfluoroalkyl substances (PFAS), PBDEs, and PCBs were evaluated for their TTR-binding capability. Using mass balance and steady-state assumptions, it was estimated that the median concentrations of the mixture found in maternal and infant serum can inhibit 20% of T4 binding to TTR *in vitro*; however, when translated *in vivo*, the same concentrations can only achieve 1.3% inhibition of T4 binding to TTR in maternal blood and 1.5% inhibition in infant blood (57). But Hamers et al. further argued that because of the so-called Goldilocks role of TTR in TH tissue delivery, inhibition of TH binding to TTR by EDCs may interfere with TH tissue delivery. Our simulations suggest otherwise – since a single THBP species is sufficient for the TH tissue delivery task in general, we do not expect TTR-binding EDCs will have tangible adverse impact in this context. However, given the unique transportation role of locally synthesized TTR in the brain and placenta (58–60), TTR-binding EDCs may affect TH delivery in these two tissues through interfering with local TTR.

The occupation of THBPs by EDCs essentially reduces the total amount of available THBPs and thus is not expected to affect the free THs when the exposure is constant and plasma EDC is at steady state. Many EDCs can be quickly metabolized in the body and thus have short half-lives. Daily intermittent exposure to these short-lived EDCs will lead to fluctuations of their plasma concentrations. Our simulations show that for a TBG-binding EDC of 1-h half-life and 8-h daily exposure, when its peak plasma level reaches 15 pM, i.e., the basal free T4 level, it can cause significant daily fluctuation of plasma free and tissue T4 and T3, despite their mean levels remain largely the same as when the TBG-binding EDC is absent. In comparison, a similar intermittent exposure to TTR-binding EDCs has a negligible effect. It is well known that THs have circadian rhythms, especially T3, which can oscillate with a nearly 7% peak-trough variation through the day (61). The circadian rhythm of T3 has important roles in health and its temporal correlation with TSH has been associated with longevity (62). The variation of plasma free THs induced by daily intermittent exposure to EDCs may modulate the magnitude and/or the phase of the circadian rhythm of T3, posing a health risk as a result.

### 4.5 Limitations and future directions of the minimal PBK model

Although we strived to parameterize the minimal PBK model based on our best knowledge of the production, distribution and metabolism of THs in humans, uncertainties exist for some variables and parameters. In addition to the three THBPs modeled here, a small fraction of THs is also bound to lipoproteins and certain serine proteinase inhibitors (serpins) superfamily members (63–65). It was thought that the fraction of TBG-bound T4 measured with zone electrophoresis may be overstated to some extent because of the comigration of some of these proteins, particularly in the α-globulin regions, with TBG (64). The fractions of T4 and T3 bound to these proteins can be variable and are not exactly clear. Future iterations of the PBK model may consider these binding proteins.

There is a lack of information on the free fractions (*Fu*) of T4 and T3 in the liver and other tissues. However, the specific values for these *Fu* parameters are not important for the model behaviors, because, as detailed in **Table S1**, those parameters that are associated with the clearance of free THs in *Liver* and *RB* (e.g., *k*_26_, *k*_30_, and *k*_34_ for T4 in the liver) are scaled with the *Fu* term, such that they cancel out as they are multiplied by the total tissue TH concentrations to define the reaction rates. In a modeling paper, it was estimated that free and bound T3 are 0.53 and 4.2 nM respectively in the cytosol of human liver, which produce an estimated 0.11 as the free fraction of T3 in the liver (66). In comparison, based on the *K_m_* and *V_max_* values of the liver metabolic enzymes of THs, including DIOs, SULT and UGT, the free fractions of T4 and T3 in the liver were estimated to be <0.01 (67).

Another uncertainty lies in the fraction of T4 that is converted into T3 in the *Liver* and *RB* compartments. It is believed that on average 25% of all T4 is metabolized into T3, however, how the fractions (*a*_1_ and *a*_2_ in our model) differ between tissues is unknown. In the current model, we used 25% for both *Liver* and *RB*, and as a result, 27% and 51% of T3 production occur in the two compartments respectively. This nearly 2:1 ratio is close to the 29:15 ratio estimated for T3 production in muscle and liver in euthyroid condition (39). Under this condition, there are net influxes of T3 from the blood to both tissue compartments (**Table 5**). In our model, in order to achieve a net efflux of T3 in *RB*, we found that *RB* must produce at least 62% of T3 and *Liver* must produce no more than 17%. It has been argued that net efflux of T3 into the circulation could occur in deiodinase 1 (DIO1) and DIO2-expressing tissues whose capacity of T3 elimination is poor (68). It is reasonable to assume that different tissues *in vivo* have different T4-to-T3 conversion and metabolism rates. Consequently, tissues with higher T4-to-T3 conversion or lower metabolism rates may contribute to the plasma T3 and those tissues with the opposite profile may deplete T3 from the plasma. But overall there is a net influx of T3 to tissues.

In the current model, we only split the whole body into the *Body Blood*, *Thyroid*, *Liver* and *RB* compartments. The lack of quantitative information on the T4 and T3 distribution and metabolism in extra-hepatic tissues prevented us from further splitting *RB* into kinetically distinct compartments such as fast and slow pools for T4 and T3 turnover (23). The liver blood flow in our model represents the total flow from both the hepatic artery and portal vein, where the hepatic blood flow only accounts for about 20%. The *RB* compartment here in theory does not contain the organs draining into the portal vein. But because the gastrointestinal track, spleen, pancreas, and esophagus add up only to slightly over 2% of total body weight, the exclusion of these organs from *RB* should only have a negligible effect on the model kinetics.

Our model does not include the HPT negative feedback loop. This exclusion, however, does not affect the analyses and conclusions for all the steady-state behaviors of the TH kinetics. Even for the TH tracer simulations, the transient rise of plasma free T4 or T3 tracer only lasts for a tiny fraction of a second (simulation results not shown), thus is unlikely to induce any effect on TSH were the feedback loop included in the model. The only results that could be affected is for the intermittent exposure to THBP-binding EDCs. There, with a negative feedback of THs on TSH, the fluctuations of plasma free T4 and T3 caused by the intermittent EDC exposure may be damped to some extent but will not be completely eliminated.

Future iterations of the model may also consider individual variability in a human population using data such as NHANES which measured serum free and total T4 and T3 and TSH concentrations in individuals. However, such efforts can be challenging even for the minimal PBK model, where many parameters may be highly correlated and so there is a potential parameter identification issue.

### 4.6 Comparison with previous TH models

There is a long history of mathematical modeling of the thyroid system for both humans and other species. These models focus on various aspects of the system and their responses to perturbations. Some prime examples are summarized below. Beginning in the 1970’s, DiStefano and Chang constructed a mathematical model by using steady-state assumptions for TH and THBP binding and considering TH metabolism only in the plasma to explore the behaviors of TH tracer and the effects of thyroidectomy and T4 replacement therapy (21). Oppenheimer and Schwartz used a mathematical model to analyze the transport of T3 between plasma, cytosol and nucleus in rat liver, kidney, and other organs (22). Pilo et al. and Curti and Fresco constructed multi-compartmental models for T3 without considering plasma THBP binding and distribution based on physiological blood flow in contrast to what we did in the present study (23, 24).

Efforts have also been made to include the feedback of the HPT axis. Saratchandran et al. constructed a simple HTP feedback model without the sophistication of THBP-mediated TH transport in the plasma (69), while Liu et al. included the binding with the 3 THBPs with the assumption that the unbound THBPs are constant (70). Eisenberg et al. constructed an HPT feedback model with TH distribution in the fast and slow pools and circadian TSH oscillations to explore its clinical applications in predicting TH therapy (25). Hoermann and his colleagues used an HPT feedback model to study the function of the TSH-T3 shunt and circadian rhythm in the thyroid gland under normal (28) and primary hypothyroid conditions (71). Most recently the group has also explored the capability of a minimal HPT model to preserve free T3 homeostasis while allowing on-demand allostatic regulation (72).

Thyroid systems modeling has also been applied to understand and predict the health effects of EDCs. Kohn et al. constructed an HPT feedback model with some PBK treatment of TH distribution and metabolism in rats and used the model to explore the disruption of TH metabolism by dioxin (73). Mclanahan et al. used a simple PK model for THs with feedback inhibition of TSH to explore the effects of iodine deficiency and sodium/iodide symporter (NIS) inhibitor perchlorate on TH levels (26, 74). Willemin and Lumen used a similar model without TSH feedback to study another NIS inhibitor thiocyanate’s effect on TH levels in rats (75). Lumen and her colleagues also used a sophisticated iodine PBPK model coupled with simple PK models for THs to explore the effects of iodine deficiency and perchlorate during pregnancy in women (27, 76). Hisham and colleagues have constructed rodent HPT axis models to understand and predict the thyroid health outcomes of environmental chemicals in combination with *in vitro* toxicity testing assays (29, 77, 78). None of these models have considered the THBP binding, if any, in as much detail as we did here, the inclusion of which will help achieve refined predictions for thyroid disrupting chemicals.

## 5 Conclusion

In summary, we have constructed a minimal human PBK model with explicit considerations of TH and THBP interactions and explored their roles in the kinetics of THs and assessing the impact of EDCs. The model provides several novel insights into the roles of THBPs and effects of EDCs that may be experimentally validated in future. Future iterations of the model may consider to include division into more tissue compartments relevant to TH kinetics, other TH-binding proteins, individual variability, HPT feedback, and circadian rhythm.

## Data availability statement

The original contributions presented in the study are included in the article/**Supplementary Material**. Further inquiries can be directed to the corresponding author.

## Author contributions

QZ conceived the model structure. AB constructed and simulated the model in MATLAB. AB and QZ conducted the parameter justification and estimation, and formal analysis of simulation results. AB and QZ wrote the initial draft and revised the manuscript. BJ critically reviewed and revised the manuscript. All authors contributed to the article and approved the submitted version.
