# Supplementary material for: A minimal human physiologically based kinetic model of thyroid hormones and chemical disruption of plasma thyroid hormone binding proteins
Source: Front Endocrinol (Lausanne). 2023 May 25;14:1168663. doi: 10.3389/fendo.2023.1168663 (PMC10248451; doi:10.3389/fendo.2023.1168663)
Supplement: Supplementary file 1 [file Image_1.pdf]

Supplemental Figures

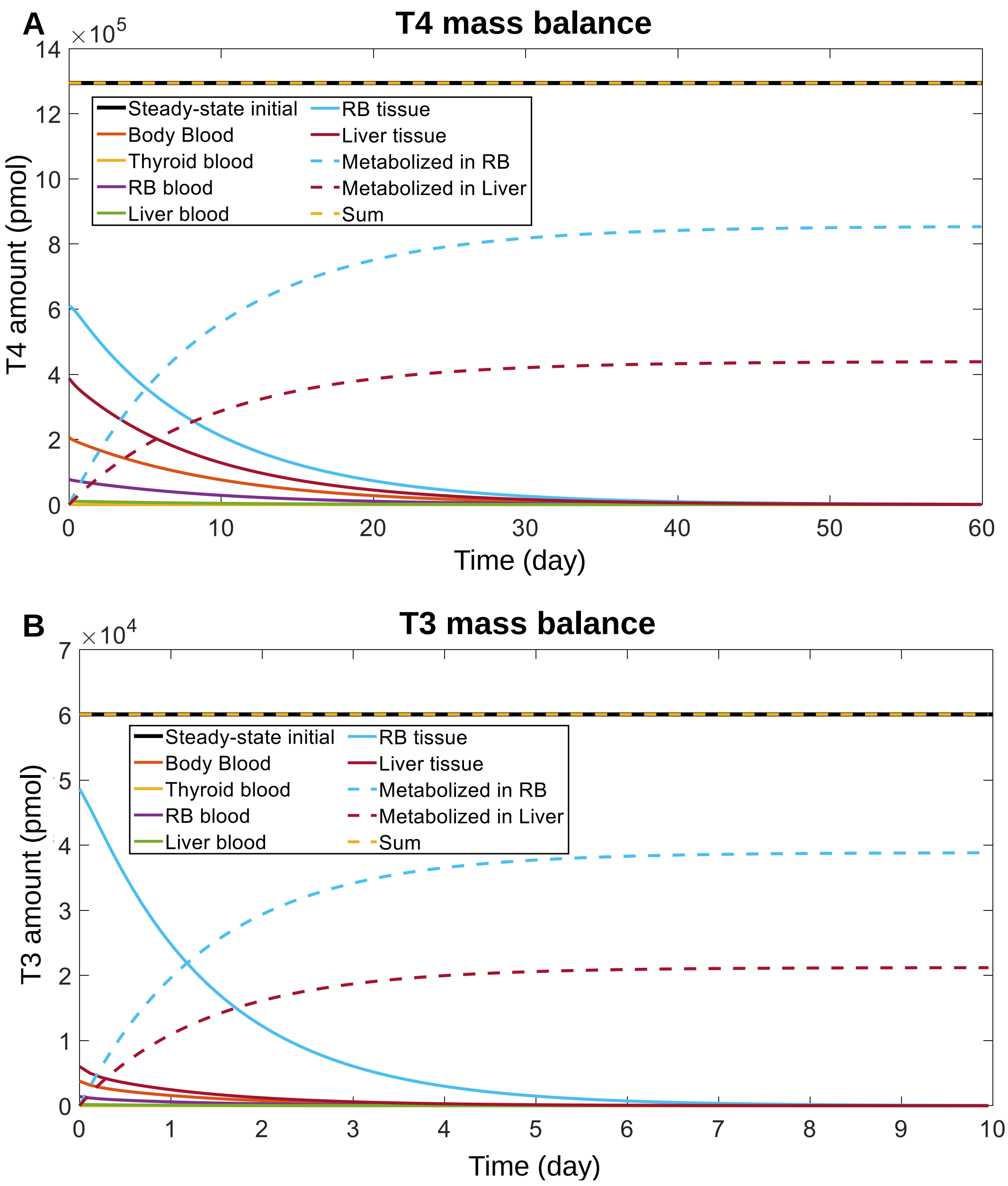

Figure S1. Mass balance check for the PBK model. (A) For T4 mass balance check, after the model is run to steady state, T4 production is terminated by setting k_{20}=0, then the amounts of T4 in various compartments (solid line) and metabolized (dashed line) as indicated are tracked, and the sum of which (yellow dashed) is compared with the steady-state initial T4 amount (black solid) before setting k_{20}=0. **(B)** For T3 mass balance check, after the model is run to steady state, T3 production is terminated by setting k_{20}=k_{24}=k_{26}=0, then the amounts of T3 in various compartments (solid line) and metabolized (dashed line) as indicated are tracked, and the sum of which (yellow dashed) is compared with the steady-state initial T4 amount (black solid) before setting k_{20}=k_{24}=k_{26}=0.

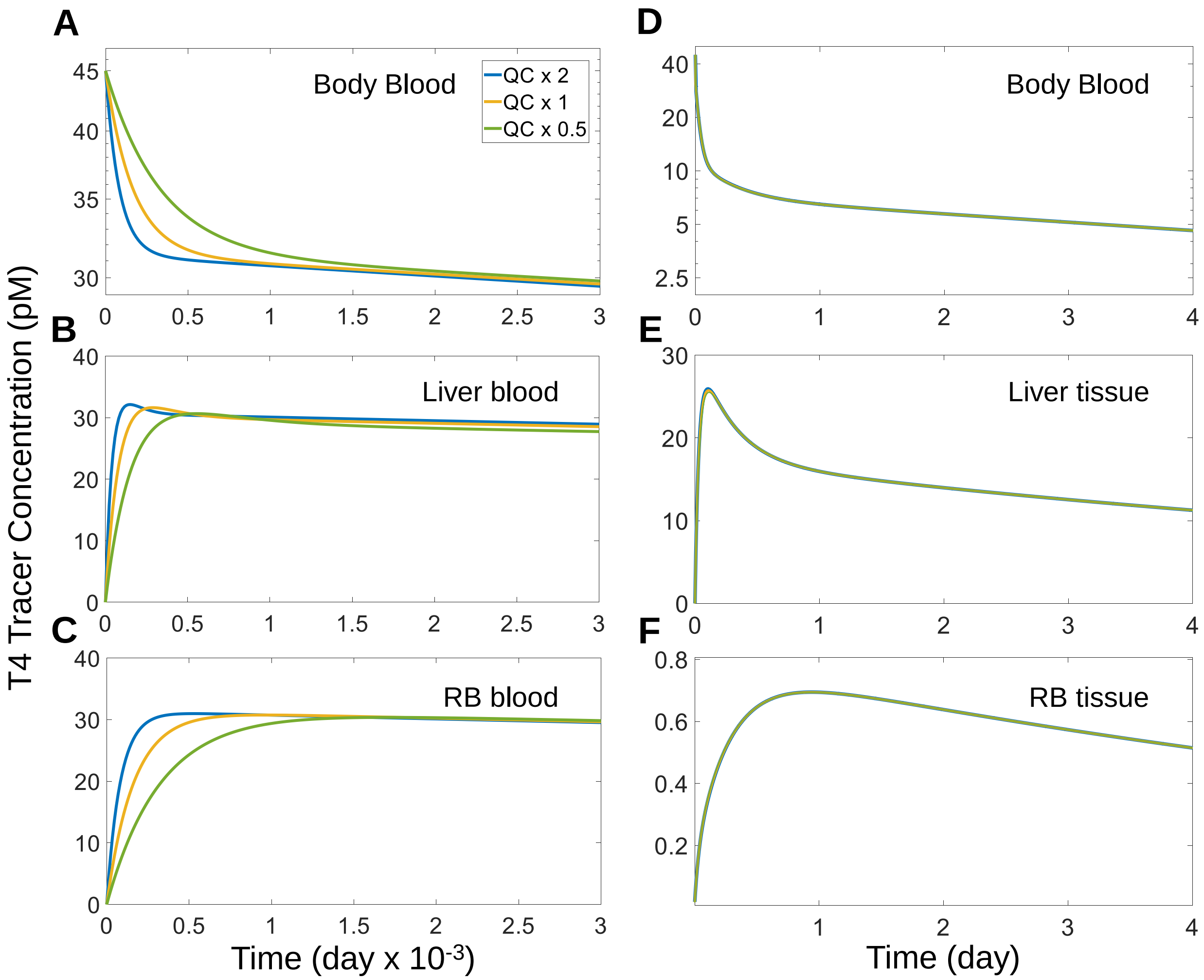

Figure S2. Effects of blood flow rate on T4 distribution kinetics. For the T4 tracer simulation, 45 pM of *fT*4 tracer is added to the *Body Blood* compartment at time 0. Plasma concentrations of total T4 tracer in *Body Blood* **(A)**, *Liver blood* **(B)**, and *RB blood* **(C)** over 0.003 day (about 4.32 min). Concentrations of plasma total T4 tracer in *Body Blood* **(D)**, T4 tracer in *Liver tissue* **(E)**, and in *RB tissue* **(F)** over 4 days. Cardiac output QC is varied to 2 or 0.5-fold of the default value as indicated in (A).

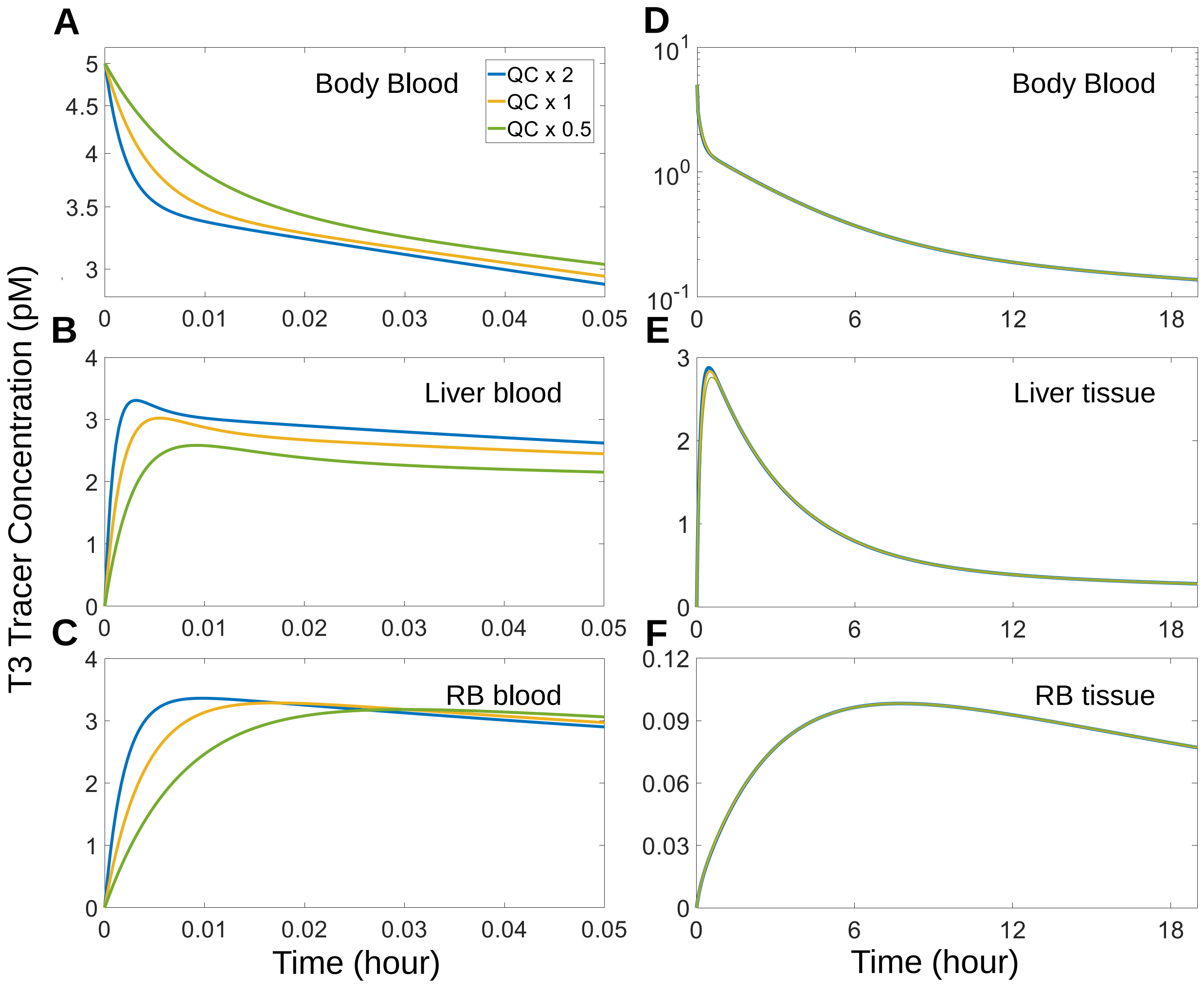

Figure S3. Effects of blood flow rate on T3 distribution kinetics. For the T3 tracer simulation, 5 pM of *fT*3 tracer is added to the *Body Blood* compartment at time 0. Plasma concentrations of total T3 tracer in *Body Blood* **(A)**, *Liver blood* **(B)**, and *RB blood* **(C)** over 0.05 hour (3 min). Concentrations of plasma total T3 tracer in *Body Blood* **(D)**, T3 tracer in *Liver tissue* **(E)**, and in *RB tissue* **(F)** over 19 days. Cardiac output QC is varied to 2 or 0.5-fold of the default value as indicated in (A).

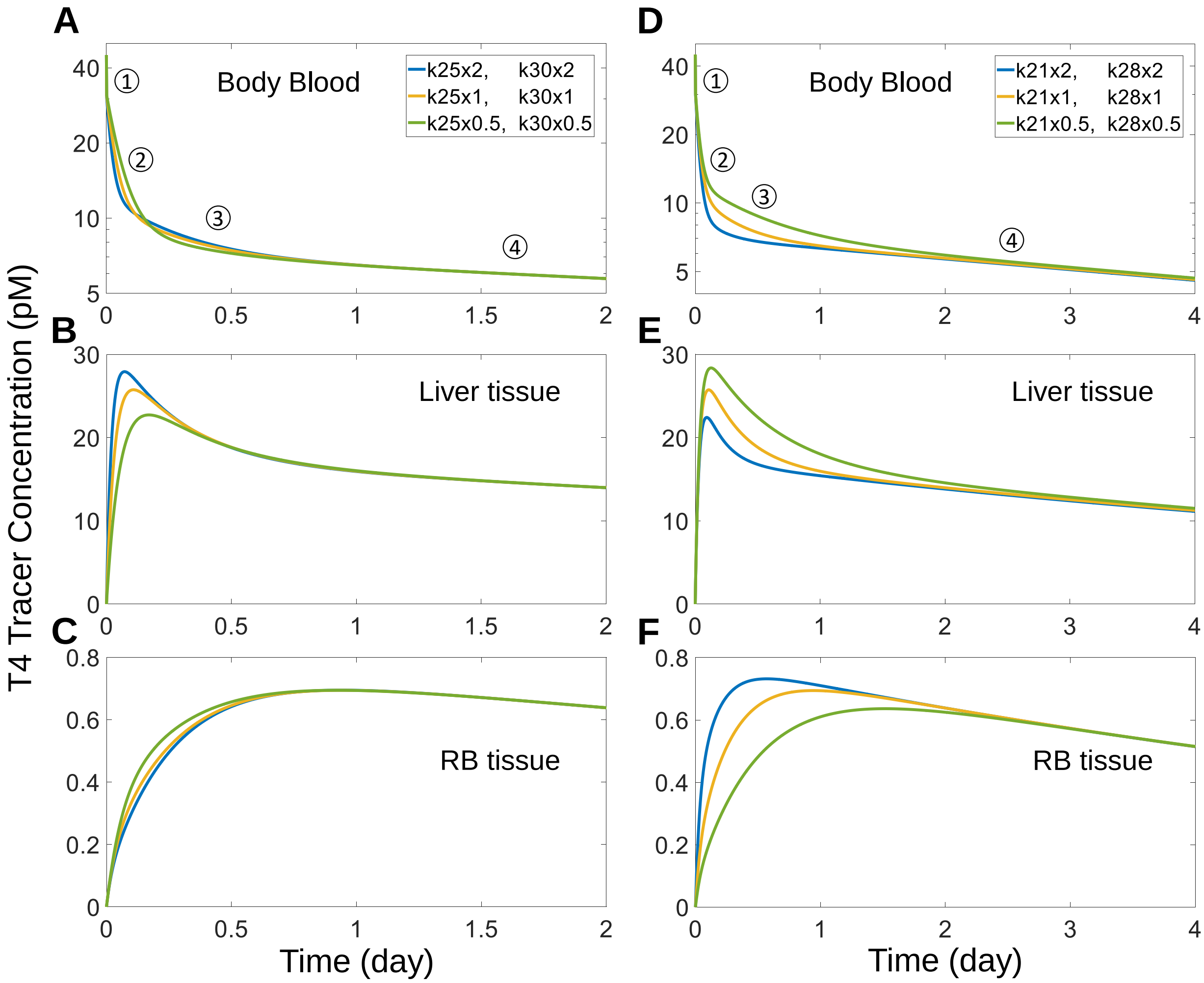

Figure S4. Effects of influx and efflux in *Liver* and *RB* on T4 distribution kinetics. For the T4 tracer simulation, 45 pM of *f*T4 tracer is added to the *Body Blood* compartment at time 0. Concentrations of T4 tracer in *Body Blood* **(A)**, *Liver tissue* **(B)**, and *RB tissue* **(C)** over time with k_{25} and k_{30} simultaneously varied to 2 or 0.5-fold of the default values as indicated in (A). Concentrations of T4 tracer in *Body Blood* **(D)**, *Liver tissue* **(E)**, and *RB tissue* **(F)** over time with k_{21} and k_{28} simultaneously varied to 2 or 0.5-fold of the default values as indicated in (D). Circled numbers in (A) and (D) indicate the four phases of plasma T4 tracer clearance.

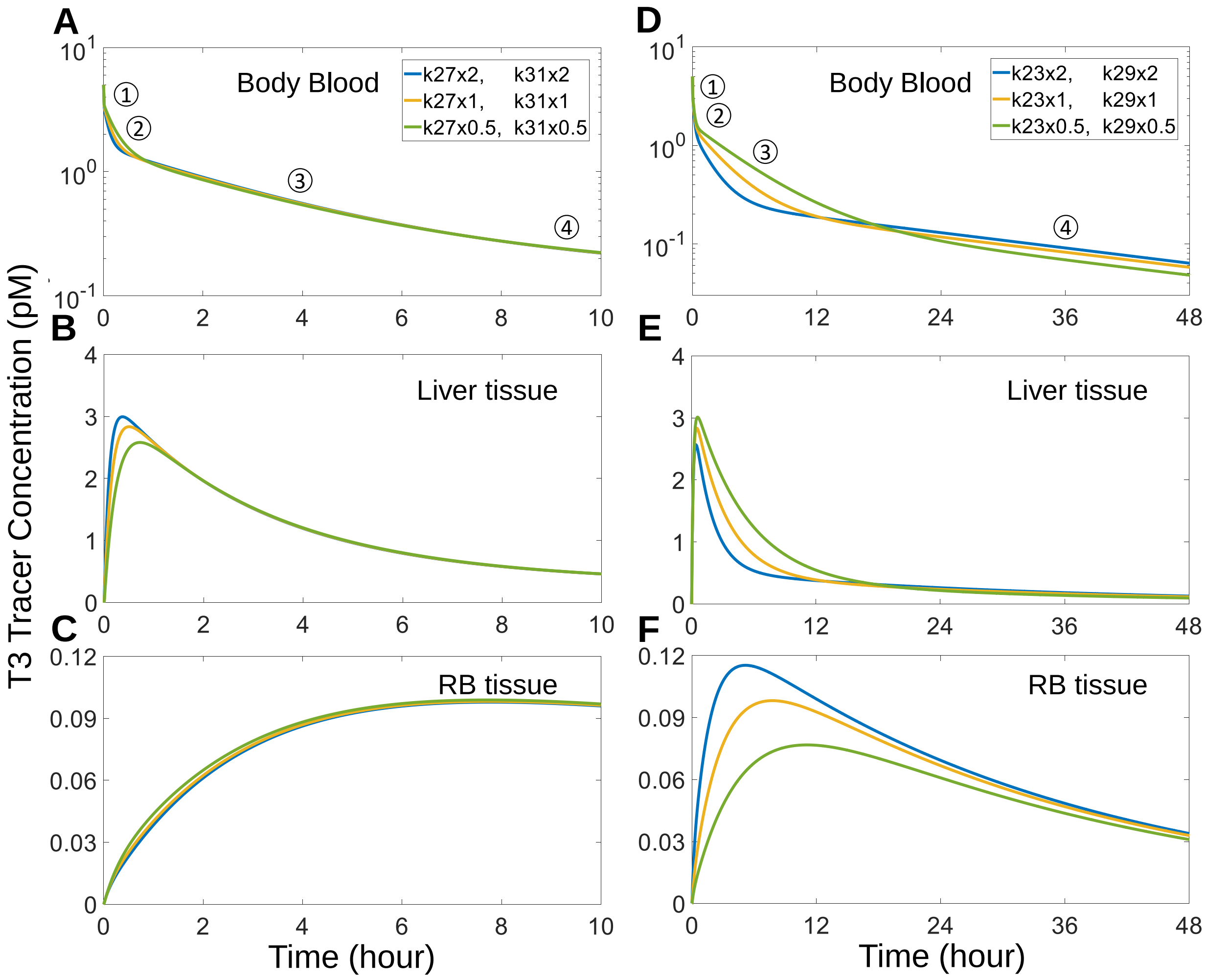

Figure S5. Effects of influx and efflux in *Liver* and *RB* on T3 distribution kinetics. For the T3 tracer simulation, 5 pM of *fT*3 tracer is added to the *Body Blood* compartment at time 0. Concentrations of T3 tracer in *Body Blood* **(A)**, *Liver tissue* **(B)**, and *RB tissue* **(C)** over time with k_{27} and k_{31} simultaneously varied to 2 or 0.5-fold of the default values as indicated in (A). Concentrations of T3 tracer in *Body Blood* **(D)**, *Liver tissue* **(E)**, and *RB tissue* **(F)** over time with k_{23} and k_{29} simultaneously varied to 2 or 0.5-fold of the default values as indicated in (D). Circled numbers in (A) and (D) indicate the four phases of plasma T3 tracer clearance.

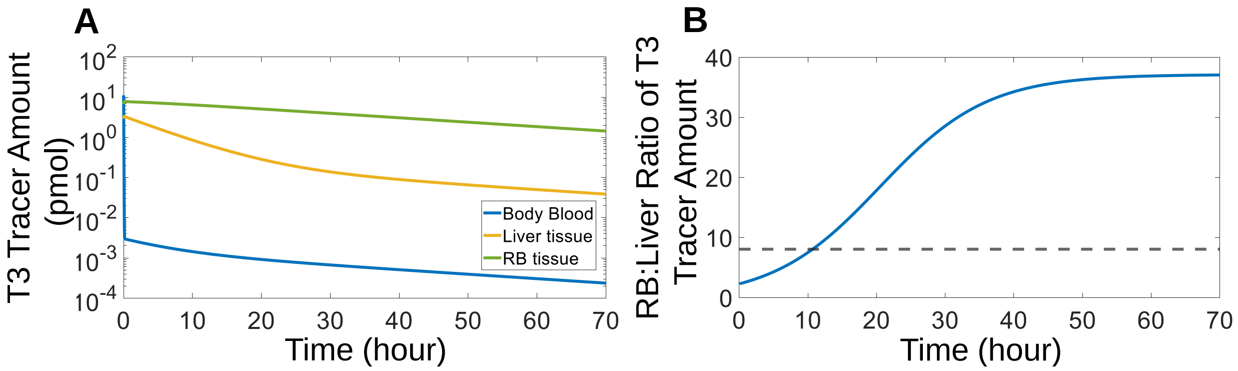

Figure S6. Increased *RB tissue*:*Liver tissue* ratio of T3 tracer amounts and lengthened T3 half-life in the <u>absence</u> of all three THBPs. 5 pM of *fT*3 tracer is added to the *Body Blood* compartment at time 0. **(A)** Amounts of T3 tracer over time in compartments as indicated. **(B)** *RB tissue*:*Liver tissue* ratio of T3 tracer amounts (blue line); *RB tissue*:*Liver tissue* ratio of steady-state endogenous T3 amounts (dashed line). In the absence of THBPs, T3 tracer is quickly distributed into *RB* and *Liver* to establish a quasi-steady state initially with an initial abundance ratio of 2.3:1. Since the T3 metabolism rate constant k_{35} in *Liver* is about 5.4 times greater than the metabolism rate constant k_{33} in *RB*, T3 tracer in *Liver* drops faster, drawing T3 tracer from the blood and in turn from *RB*, thus promoting T3 tracer clearance from *RB*. This process continues until a constant *RB tissue*:*Liver tissue* ratio of T3 tracer amounts is reached, which is 37:1 here. Thereafter, T3 trace in both tissues drop with the same logarithmic slope. Given the predominant partitioning of T3 tracer in *RB* than in *Liver*, the overall half-life is 27.25 h, which is close to the intrinsic T3 half-life of 30.39 h in *RB tissue* as determined by k_{33}.

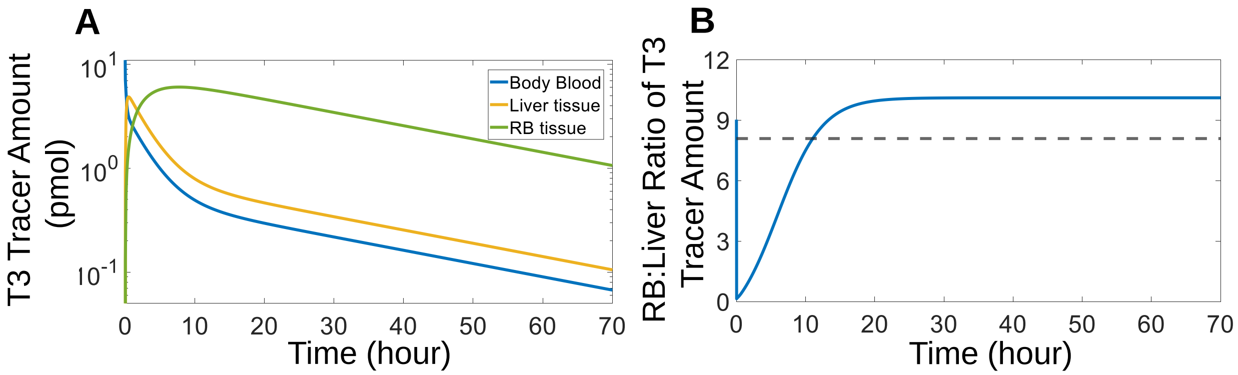

Figure. S7. *RB tissue*:*Liver tissue* ratio of T3 tracer amounts in the <u>presence</u> of all three THBPs. 5 pM of *fT*3 tracer is added to the *Body Blood* compartment at time 0. **(A)** Amounts of T3 tracer over time in compartments as indicated. **(B)** *RB tissue*:*Liver tissue* ratio of T3 tracer amounts (blue line); *RB tissue*:*Liver tissue* ratio of steady-state endogenous T3 amounts (dashed line).

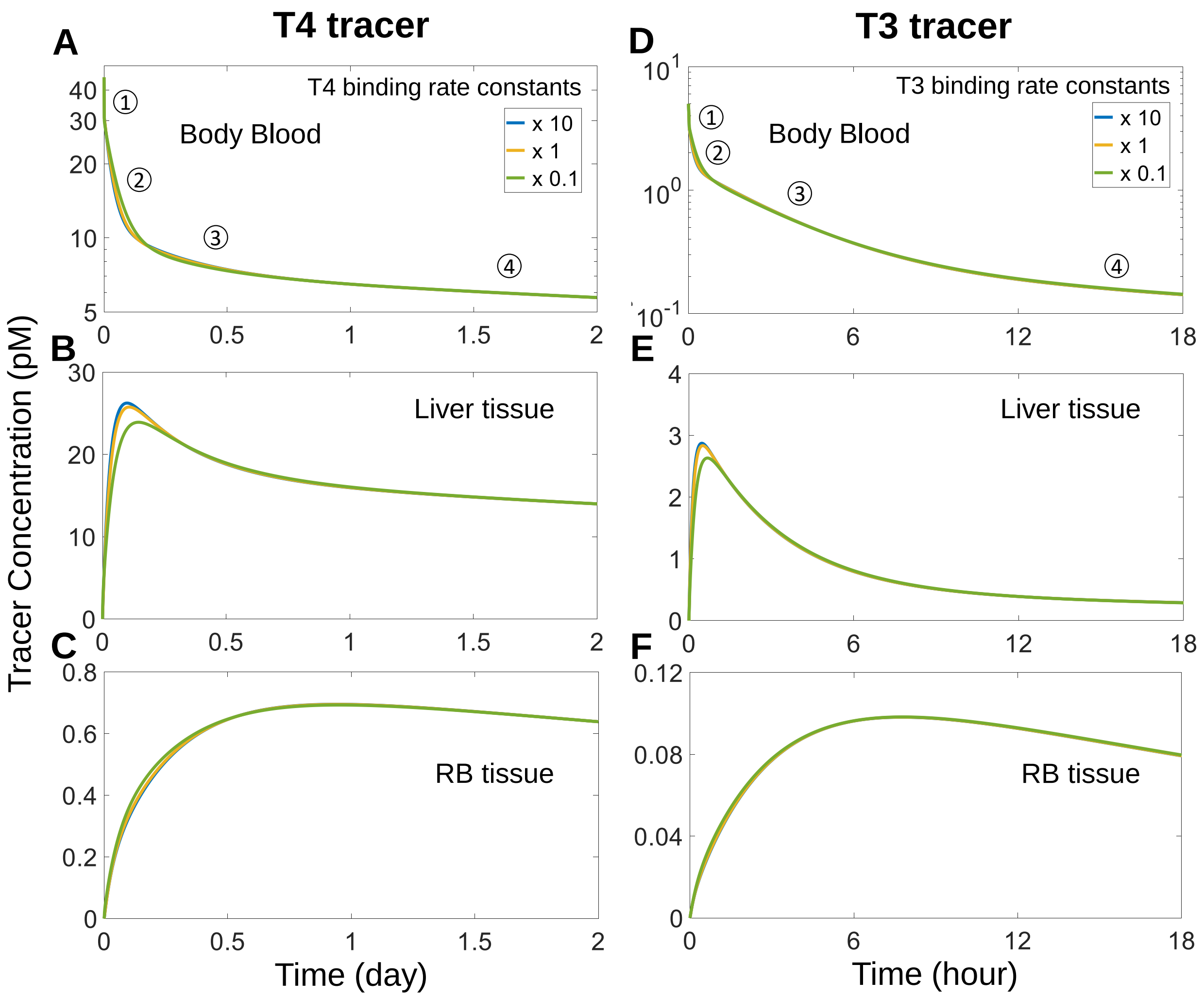

Figure S8. Effects of THBP binding rates on TH distribution kinetics. (A-C) For T4 tracer experiment, 45 pM of *fT*4 tracer is added to the *Body Blood* compartment at time 0. Concentrations of T4 tracer in *Body Blood* **(A)**, *Liver tissue* **(B)**, and *RB tissue* **(C)** over time with k_1 and k_2, k_3 and k_4, and k_5 and k_6 simultaneously varied by 10-fold or 0.1-fold relative to the default values as indicated in (A). **(D-F)** For T3 tracer experiment, 5 pM of *fT*3 tracer is added to the *Body Blood* compartment at time 0. Concentrations of T3 tracer in *Body Blood* **(A)**, *Liver tissue* **(B)**, and *RB tissue* **(C)** over time with k_7 and k_8, k_9 and k_{10}, and k_{11} and k_{12} simultaneously varied by 10-fold or 0.1-fold relative to the default values as indicated in (D). Circled numbers in (A) and (D) indicate the four phases of T4 and T3 tracer clearance respectively.

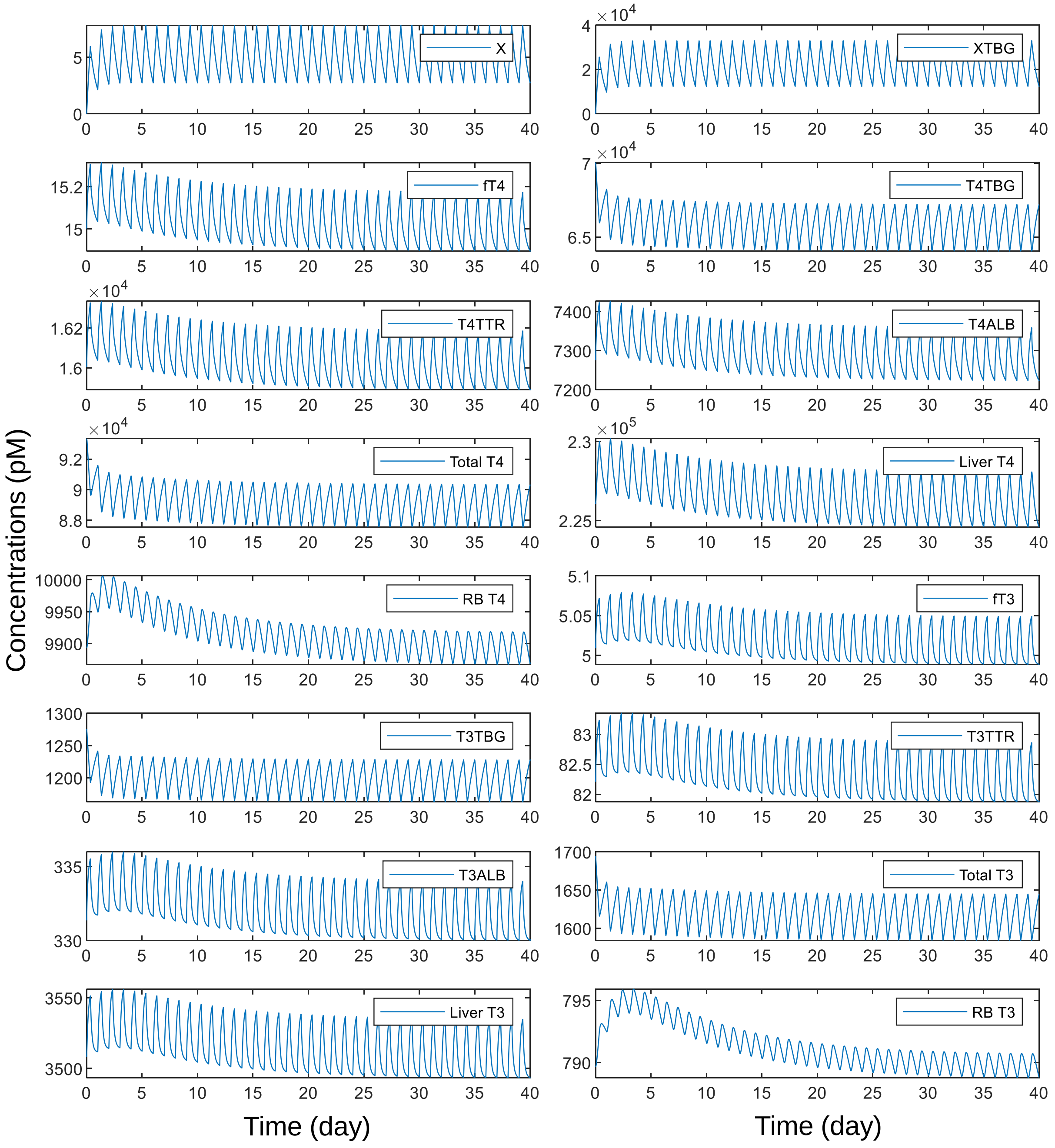

Figure S9. Time-course responses to 8-h daily exposure to TBG-binding EDC *X* that has a half-life of **10 h** in the plasma. The exposure level is set such that if the exposure is continuous for 24 h each day, it produces a steady-state free plasma *X* concentration of 15 pM. *X* binds to TBG with the same affinity and rate constants as T4.

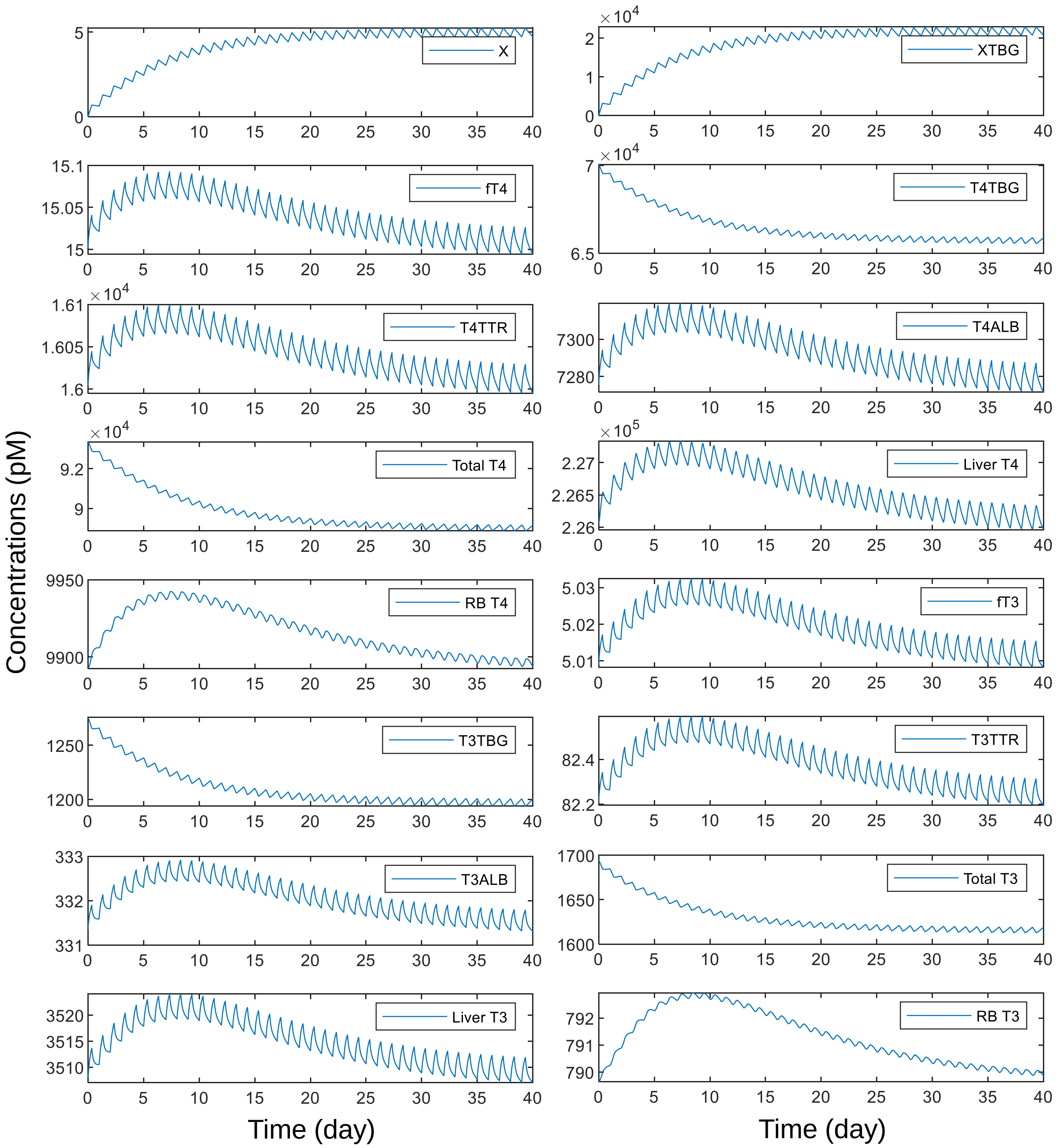

Figure S10. Time-course responses to 8-h daily exposure to TBG-binding EDC *X* that has a half-life of **100 h** in the plasma. The exposure level is set such that if the exposure is continuous for 24 h each day, it produces a steady-state free plasma *X* concentration of 15 pM. *X* binds to TBG with the same affinity and rate constants as T4.

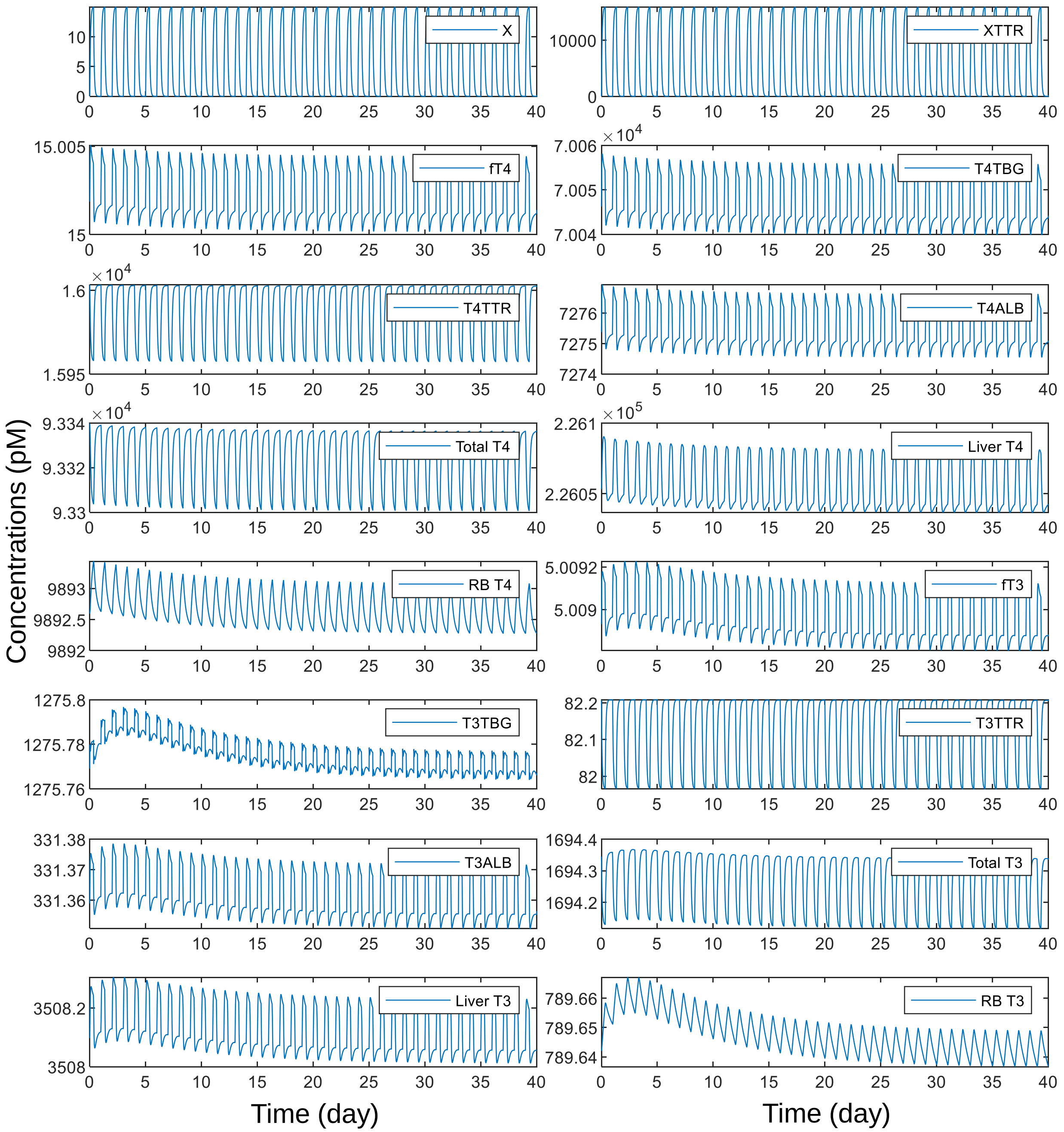

Figure S11. Time-course responses to 8-h daily exposure to TTR-binding EDC *X* that has a half-life of **1 h** in the plasma. The exposure level is set such that if the exposure is continuous for 24 h each day, it produces a steady-state free plasma *X* concentration of 15 pM. *X* binds to TTR with the same affinity and rate constants as T4.

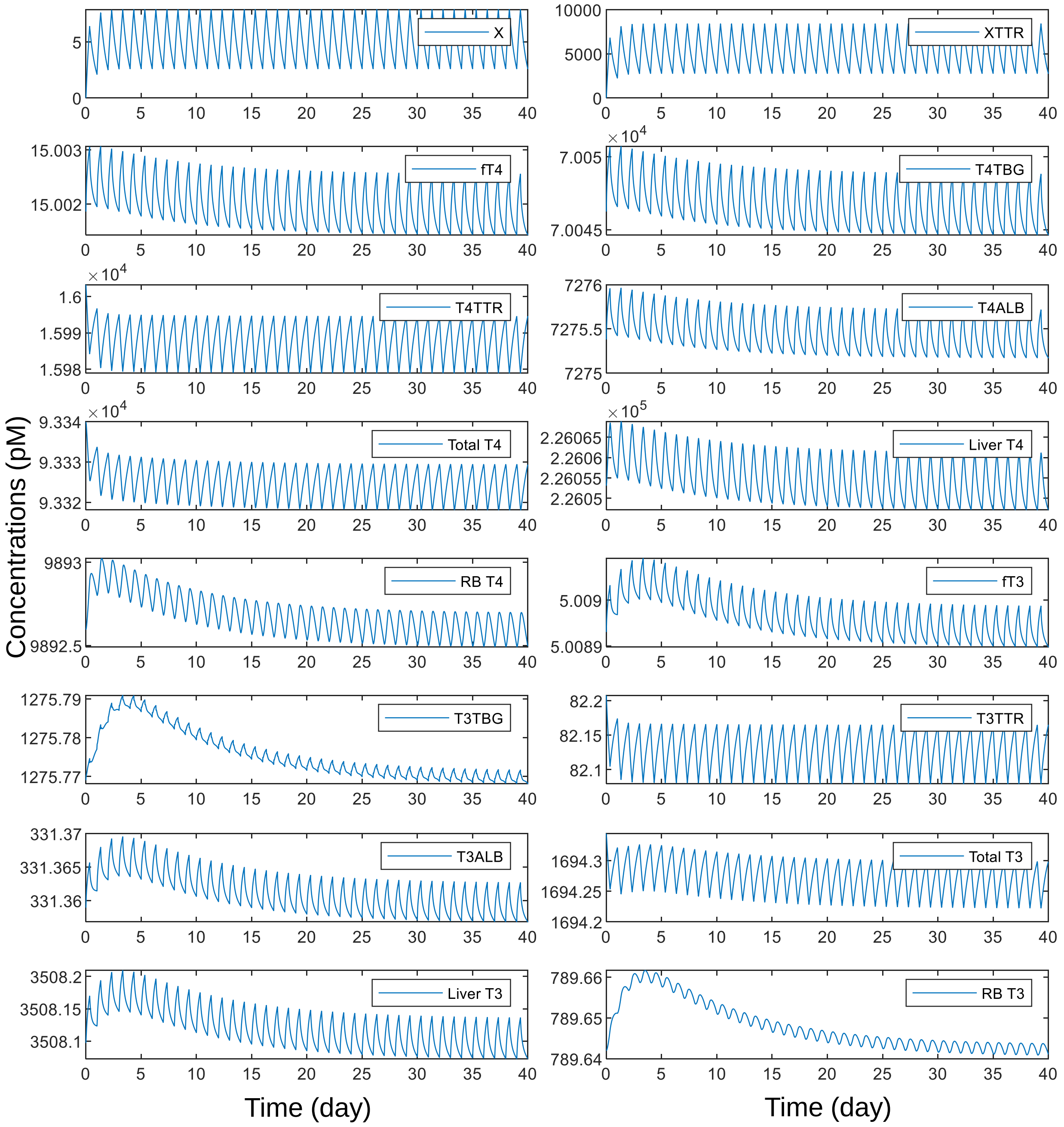

Figure S12. Time-course responses to 8-h daily exposure to TTR-binding EDC *X* that has a half-life of **10 h** in the plasma. The exposure level is set such that if the exposure is continuous for 24 h each day, it produces a steady-state free plasma *X* concentration of 15 pM. *X* binds to TTR with the same affinity and rate constants as T4.

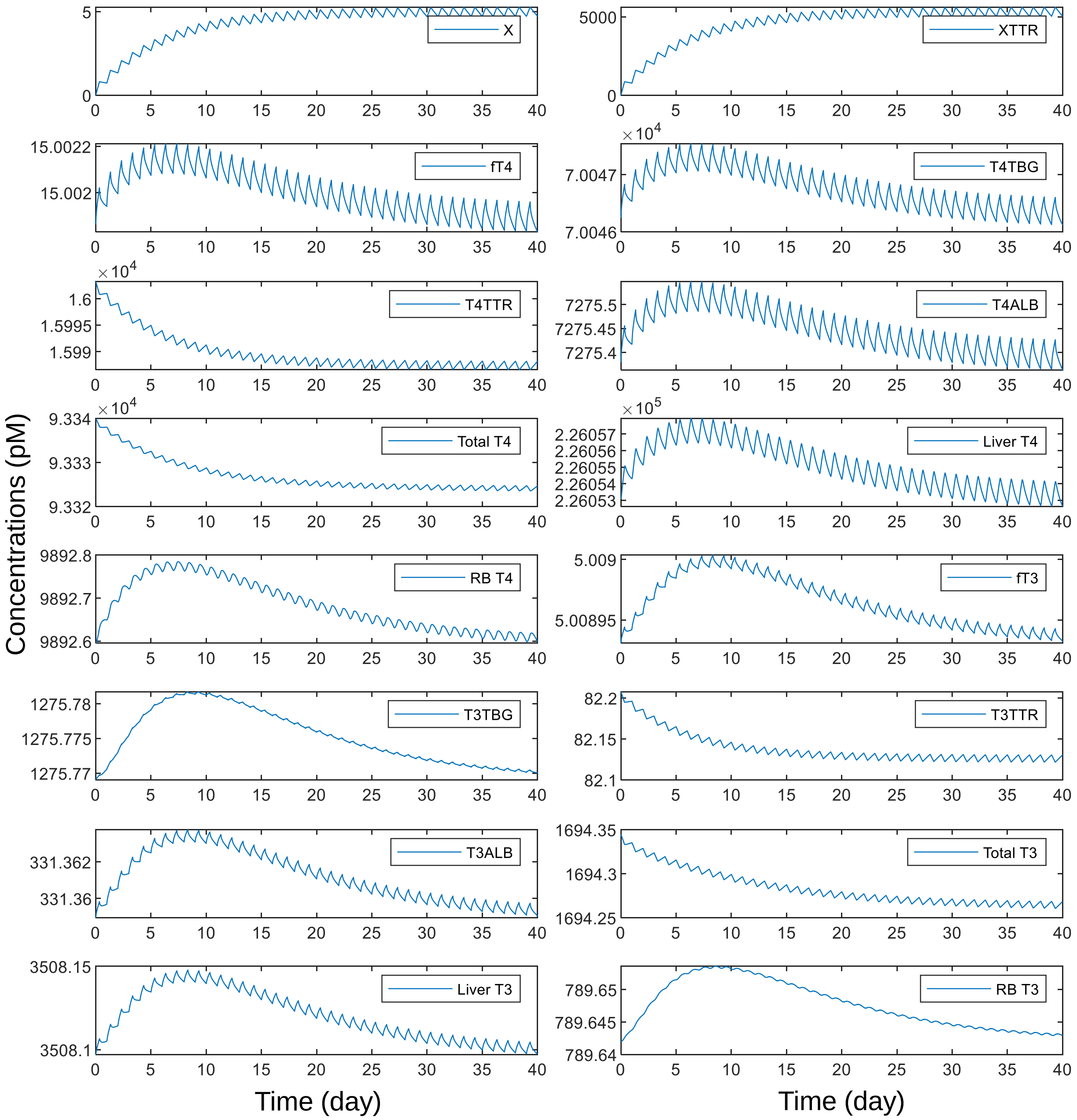

Figure S13. Time-course responses to 8-h daily exposure to TTR-binding EDC *X* that has a half-life of **100 h** in the plasma. The exposure level is set such that if the exposure is continuous for 24 h each day, it produces a steady-state free plasma *X* concentration of 15 pM. *X* binds to TTR with the same affinity and rate constants as T4.
