# Supplementary material for: A minimal human physiologically based kinetic model of thyroid hormones and chemical disruption of plasma thyroid hormone binding proteins
Source: Front Endocrinol (Lausanne). 2023 May 25;14:1168663. doi: 10.3389/fendo.2023.1168663 (PMC10248451; doi:10.3389/fendo.2023.1168663)
Supplement: Supplementary file 2 [file Table_1.pdf]

Table S1. Model parameter values, references and justifications

Parameter	Symbol	Value	Unit	Reference
Body weight	*BW*	75	kg	
Cardiac output (plasma)	*QC*	0.057 [a]	L/S	(Brown et al. 1997)
Tissue fractional blood flow of *QC*				
Thyroid	*QTC*	0.015	-	(Brown et al. 1997)
Liver	*QLC*	0.25	-	(Brown et al. 1997)
RB (rest of body)	*QRBC*	0.735	-	*QRBC* = 1 - *QTC* - *QLC*
Fractional blood volume of tissue				
Thyroid	*VTBC*	0.18 [b]	-	(Brown et al. 1997)
Liver	*VLBC*	0.11	-	(Brown et al. 1997)
RB	*VRBBC*	0.0236 [c]	-	(Brown et al. 1997)
Tissue fractional volume of BW				Assuming unity density
Thyroid	*VTC*	0.0003	-	(Brown et al. 1997)
Liver	*VLC*	0.0257	-	(Brown et al. 1997)
Plasma	*VPC*	0.0424	-	(Poulin and Theil 2002)
RB	*VRBC*	0.8416	-	*VRBC* = 0.91 - *VTC* - *VLC* - *VPC*
Hematocrit	*HCT*	0.44	-	(Davies and Morris 1993)
Binding: *T4* + *TBG* ↔ *T4TBG*				
Dissociation constant	Kd_{T4TBG}	60 [d]	pM	(Murata et al. 1985, Richardson 2007, Janssen and Janssen 2017, McLean et al. 2017)
Association rate constant	k_1	3.0E-4	1/pM/S	$k_1 = k_2/Kd_{T4TBG}$
Dissociation rate constant	k_2	0.018	1/S	(Hillier 1971, Mendel et al. 1988a, Mendel and Weisiger 1990)
Binding: T4 + TTR ↔ T4TTR				
Dissociation constant	Kd_{T4TTR}	5000 [e]	pM	(Chang et al. 1999, Prapunpoj et al. 2006, Janssen and Janssen 2017, McLean et al. 2017)
Association rate constant	k_3	1.664E-5	1/pM/S	$k_3 = k_4/Kd_{T4TTR}$
Dissociation rate constant	k_4	0.0832 [f]	1/S	(Hillier 1971, Mendel et al. 1988a, Mendel and Weisiger 1990)
Binding: T4 + ALB ↔ T4ALB				
Dissociation constant	Kd_{T4ALB}	1.33E6 [g]	pM	(Barlow et al. 1982, Yabu et al. 1987, Petitpas et al. 2003)
Association rate constant	k_5	9.77E-7	1/pM/S	$k_5 = k_6/Kd_{T4ALB}$
Dissociation rate constant	k_6	1.3 [h]	1/S	(Mendel et al. 1988a, Mendel et al. 1990, Mendel and Weisiger 1990)
Binding: T3 + TBG ↔ T3TBG				
Dissociation constant	Kd_{T3TBG}	1100 [i]	pM	(Murata et al. 1985, Richardson 2007, Janssen and Janssen 2017, McLean et al. 2017)

Association rate constant	k_7	1.5E-4	1/pM/S	$k_7 = k_8/Kd_{T3TBG}$
Dissociation rate constant	k_8	0.165 [j]	1/S	(Hillier 1975, Mendel et al. 1988b)
Binding: T3 + TTR ↔ T3TTR				
Dissociation constant	Kd_{T3TTR}	3.25E5 [k]	pM	(Chang et al. 1999, Prapunpoj et al. 2006, Janssen and Janssen 2017, McLean et al. 2017)
Association rate constant	k_9	2.1231E-6	1/pM/S	$k_9 = k_{10}/Kd_{T3TTR}$
Dissociation rate constant	k_{10}	0.69 [l]	1/S	(Mendel et al. 1988b, Mendel 1989, Richardson 2007)
Binding: T3 + ALB ↔ T3ALB				
Dissociation constant	Kd_{T3ALB}	9.75E6 [m]	pM	(Yabu et al. 1987, Richardson 2007, Janssen and Janssen 2017)
Association rate constant	k_{11}	2.256E-7	1/pM/S	$k_{11} = k_{12}/Kd_{T3ALB}$
Dissociation rate constant	k_{12}	2.2 [n]	1/S	(Hillier 1975, Mendel et al. 1990)
Thyroidal T4 production rate	k_{20}	1.6 [o]	pmol/S	(Chopra 1976, Pilo et al. 1990)
Rate constant for RB tissue T4 influx	k_{21}	1 [p]	L/S	Calculated
Thyroidal T3 production rate	k_{22}	0.1143 [q]	pmol/S	$k_{22} = k_{20}/14 = 0.1143$ (Pilo et al. 1990)
Rate constant for *RB Tissue* T3 influx	k_{23}	0.15 [r]	L/S	Calculated
Rate constant for T4→T3 conversion in *RB Tissue*	k_{24}	$1.7084E\text{-}6/fu_{T4RBT}*a_2$ [s]	1/S	Calculated
Rate constant for *Liver Tissue* T4 influx	k_{25}	4.243 [t]	L/S	Calculated
Rate constant for T4→T3 conversion in liver	k_{26}	$1.44E\text{-}6/fu_{T4LT}*a_1$ [u]	1/S	Calculated
Rate constant for *Liver Tissue* T3 influx	k_{27}	1.398 [v]	L/S	Calculated
Rate constant for *RB Tissue* T4 efflux	k_{28}	$0.00141/fu_{T4RBT}$ [w]	L/S	Calculated
Rate constant for *RB Tissue* T3 efflux	k_{29}	$8.9E\text{-}4/fu_{T3RBT}$ [x]	L/S	Calculated
Rate constant for *Liver Tissue* T4 efflux	k_{30}	$2.786\text{-}4/fu_{T4LT}$ [y]	L/S	Calculated
Rate constant for *Liver Tissue* T3 efflux	k_{31}	$1.9688E\text{-}3/fu_{T3LT}$ [z]	L/S	Calculated
Rate constant for T4 metabolism excluding conversion to T3 in *RB Tissue*	k_{32}	$1.7084E\text{-}6/fu_{T4RBT}*(1-a_2)$ [aa]	1/S	Calculated
Rate constant for T3 metabolism in *RB Tissue*	k_{33}	$6.3349E\text{-}6/fu_{T3RBT}$ [bb]	1/S	Calculated
Rate constant for T4 metabolism excluding conversion to T3 in *Liver Tissue*	k_{34}	$1.44E\text{-}6/fu_{T4LT}*(1-a_1)$ [cc]	1/S	Calculated

Rate constant for T3 metabolism in *Liver Tissue*	k_{35}	3.4229E-5 / fu_{T3LT} [dd]	1/S	Calculated
Fraction of T4 converted to T3 in *Liver Tissue*	a_1	0.25 [ee]	-	(Chopra 1976, Pilo et al. 1990, Maia et al. 2005)
Fraction of T4 converted to T3 in *RB Tissue*	a_2			
Free fraction of T4 in *Liver Tissue*	fu_{T4LT}	< 1 [ff]	-	(Pardridge and Landaw 1987)
Free fraction of T4 in *RB Tissue*	fu_{T4RBT}			
Free fraction of T3 in *Liver Tissue*	fu_{T4LT}			
Free fraction of T3 in *RB Tissue*	fu_{T4RBT}			
Total concentration of plasma TBG	TBG_{tot}	3.515E5 [gg]	pM	(Attwood et al. 1978, Attwood and Atkin 1982, Franklyn and Shepard 2000, Schussler 2000, Richardson 2007, Janssen and Janssen 2017, McLean et al. 2017)
Total concentration of plasma TTR	TTR_{tot}	5.35E6 [hh]	pM	(Vatassery et al. 1991, Franklyn and Shepard 2000, Schussler 2000, Richardson 2007, Janssen and Janssen 2017, McLean et al. 2017)
Total concentration of plasma ALB	ALB_{tot}	6.45E8 [ii]	pM	(Gardner and Scott 1980, Barlow et al. 1982, Schussler 2000, Richardson 2007, Janssen and Janssen 2017, McLean et al. 2017)

Table S2. Values and ranges used for parameter estimation and model validation

Metric Name	Value or Range	Unit	References
% of plasma total T4 as T4TBG	74%-75%	-	(Schussler 2000, Janssen and Janssen 2017, McLean et al. 2017)
% of plasma total T4 as T4TTR	11%-20%	-	(Schussler 2000, Janssen and Janssen 2017, McLean et al. 2017)
% of plasma total T4 as T4ALB	5%-15%	-	(Schussler 2000, Janssen and Janssen 2017, McLean et al. 2017)
% of plasma total T3 as T3TBG	75%	-	(Janssen and Janssen 2017)
% of plasma total T3 as T3TTR	<5%	-	(Janssen and Janssen 2017)
% of plasma total T3 as T3ALB	<20%	-	(Janssen and Janssen 2017)
% of plasma total T4 as free T4	0.005%-0.03% [jj]	-	(Mendel 1989, Schussler 2000, Richardson 2007, McLean et al. 2017)
% of plasma total T3 as free T3	0.3%-0.4%	-	(Mendel 1989, Richardson 2007, McLean et al. 2017)
% saturation of plasma total TBG by T4	18.4%-20%	-	(Schussler 2000, McLean et al. 2017)
% saturation of plasma total TTR by THs	0.16%-0.5%	-	(Schussler 2000, McLean et al. 2017)
% saturation of plasma total ALB by THs	0.0016%	-	(Schussler 2000)
fT4	15 [kk]	pM	(Franklyn and Shepard 2000, Jain 2015)
fT3	5 [ll]	pM	(Franklyn and Shepard 2000, Jain 2015)
Total T4	Mean: 9.62E4, 9.79E4<br>Range: 5.79E4-16.1E4 [mm]	pM	(Franklyn and Shepard 2000, Aoki et al. 2007, Jain 2015)
Total T3	Mean: 1.772E3<br>Range: 1.2E3-3.08E3 [nn]	pM	(Franklyn and Shepard 2000, Aoki et al. 2007)
Free T3 in liver	530, 1000 [oo]	pM	(Pardridge and Landaw 1987, Mendel 1989)
% distribution of T4 in extrathyroidal tissues			
Blood	28.8%	-	(Pilo et al. 1990)
Liver Tissue (*AT4LTC*)	30% [pp]	-	(Cavalieri and Searle 1966, Felicetta et al. 1980)
Liver:plasma T4 amount ratio	1.3-1.4 (median), 0.97-1.85	-	(Felicetta et al. 1980)
% distribution of T3 in extrathyroidal tissues			
Blood	7.7% or <10%	-	(Cavalieri et al. 1970, Pilo et al. 1990)
Liver Tissue (*AT3LTC*)	10% [qq]	-	(Cavalieri et al. 1970, Pilo et al. 1990, Curti and Fresco 1992)
T3 production rate in liver	12 [rr]	nmol/75 kg BW/day	(Maia et al. 2005)
T3 production rate in muscle	23.3 [ss]	nmol/75 kg BW/day	(Maia et al. 2005)

Total T3:T4 molar production ratio	0.37, 0.38 [tt]	-	(Nicoloff et al. 1972, Chopra 1976)
% of total T3 production in extrathyroidal tissue	76.2%	-	(Chopra 1976)
T4 half-life ($t_{1/2T4}$)	6.5 [uu]	Day	(Sterling and Chodos 1956, Cavalieri and Searle 1966, Nicoloff et al. 1972)
T3 half-life ($t_{1/2T3}$)	22.5 [vv]	Hour	(Cavalieri et al. 1971, Nicoloff et al. 1972, Jonklaas et al. 2015)
% T4 metabolized by *Liver Tissue* (*MT4LTC*)	35% [ww]	-	(Mendel et al. 1988a)
% T4 liver blood supply rate as unidirectional uptake rate (influx) by *Liver Tissue* (*IT4LC*)	4.8% [xx]	-	(Mendel 1989)
% of T4 and T3 taken up unidirectionally by the liver that would return to the plasma unmetabolized	>99%	-	(Mendel 1989)
% T3 liver blood supply rate as unidirectional uptake rate (influx) by *Liver Tissue* (*IT3LC*)	29%	-	(Mendel 1989)
% T3 metabolized by *Liver Tissue* (*MT3LTC*)	40% [yy]	-	(Curti and Fresco 1992)

Table S3. Intermediate metric values used for parameter estimation

Metric Name	Description	Estimated Value
AT4WB	Total amount of T4 in the extrathyroidal whole body	$AT4WB = \frac{k_{20}}{(\frac{\ln(2)}{t_{1/2T4} * 24 * 3600})} = \frac{1.6}{(\frac{\ln(2)}{6.5 * 24 * 3600})} = 1.2963\text{E}6 \text{ pmol}$
AT3WB	Total amount of T3 in the extrathyroidal whole body	$AT3WB = \frac{k_{22} + k_{20} * a_1}{(\frac{\ln(2)}{t_{1/2T3} * 3600})} = \frac{\frac{1.6}{14} + 1.6 * 0.25}{(\frac{\ln(2)}{22.5 * 3600})} = 6.0099\text{E}4 \text{ pmol}$<br>Where a_1 is the fraction of T4 converted to T3 in peripheral tissues including *Liver Tissue* (a_1) and *RB Tissue* (a_2=a_1=0.25).
AT4B	Amount of T4 in all blood compartments	*AT4B* = 2.9867E5 pmol, which is obtained by clamping *fT4* in all blood compartments to 15 pM and T3 to 5 pM and summing total T4 amount in these compartments.
AT4BC	Fraction of T4 in all blood compartments	AT4BC = *AT4B* / *AT4WB* = 2.9867E5 / 1.2963E6 = 0.2304
AT3B	Amount of T3 in all blood compartments	*AT3B* = 5.3789E3 pmol, which is obtained by clamping *fT4* in all blood compartments to 15 pM and T3 to 5 pM and summing total T3 amount in these compartments.
AT3BC	Fraction of T3 in all blood compartments	AT3BC = *AT3B* / *AT3WB* = 5.3789E3 / 6.0099E4 = 0.0895
AT4LT	Amount of T4 in *Liver Tissue*	*AT4LT* = *AT4WB* * *AT4LTC* = 1.2963E6 * 0.3 = 3.8889E5 pmol
AT3LT	Amount of T3 in *Liver Tissue*	*AT3LT* = *AT3LTC* * *AT3WB* = 0.1 * 6.0099E4 = 6.0099E3 pmol
AT4RBTC	Fraction of T4 in *RB Tissue*	*AT4RBTC* = 1 - *AT4LTC* – *AT4BC* = 1 - 0.3 - 0.2304 = 0.4696
AT4RBT	Amount of T4 in *RB Tissue*	*AT4RBT* = *AT4WB* * *AT4RBTC* = 1.2963E6 * 0.4696 = 6.0874E5 pmol
AT3RBTC	Fraction of T3 in *RB Tissue*	*AT3RBTC* = 1 – *AT3LTC* – *AT3BC* = 1 - 0.1 - 0.0895 = 0.8105
AT3RBT	Amount of T3 in *RB Tissue*	*AT3RBT* = *AT3WB* * *AT3RBTC* = 6.0099E4 * 0.8105 = 4.871E4 pmol

Note for parameter estimation

[a] Calculated based on the equation QC = 15 *$BW^{0.74}$ /3600*(1-HCT) in Brown et al. 1997 for a body weight (BW) of 75 kg, where HCT is hematocrit.

[b] The value 0.18 for rats in Brown et al. 1997 is used as no human data is available.

[c] The following equation was used to calculate *VRBBC* which is a weighted average of the fractional blood volume of major tissues excluding the liver and thyroid gland: $VRBBC = \sum_{i=1}^{6}(VTissueBC_i * VTissueC_i)/\sum_{i=1}^{6} VTissueC_i$, where $i = [1, 6]$, and $VTissueBC_i$ and $VTissueC_i$ are the fractional blood volume and fractional tissue volume of BW for muscle (0.01, 0.4), fat (0.02, 0.2142), bone (0.04, 0.1429), skin (0.08, 0.0371), brain (0.04, 0.02), and kidney (0.36, 0.0044) reported in Brown et al. 1997, respectively.

[d] The review papers by Richardson 2007, Janssen and Janssen 2017, McLean et al. 2017 provided a value of 100 pM. Murata et al. 1985 reported 100 and 92 pM for Caucasians and Australian aborigines respectively. The Murata paper also summarized a number of studies reporting values ranging from 42.5 to 400 pM. In our model, Kd_{T4TBG} value is set to 60 pM such that *T4TBG* is about 75% of total plasma T4.

[e] The review papers by Richardson 2007 and McLean et al. 2017 provided a value of 5,000 and 14,286 pM respectively. Prapunpoj et al. 2006 reported a value of 19,730 pM. Chang et al. 1999 reported a value of 13,600 pM. The Chang paper also summarized a number of studies reporting values ranging from 300 to 1.28E5 pM. In our model Kd_{T4TTR} is set to 5,000 pM such that *T4TTR* is about 17% of total plasma T4.

[f] The reported values are 0.0877, 0.08, and 0.082 S^{-1} in Hillier 1971, Mendel et al. 1988, and Mendel and Weisiger 1990 respectively. The average value 0.0832 is used in our model.

[g] The reported values are 4.3E6, 4.7619E5, and 2.3E6 pM in Barlow et al. 1982, Yabu et al. 1987, and Petitpas et al. 2003 respectively. In our model Kd_{T4ALB} is set to 1.33E6 pM (which falls in the above range) such that *T4ALB* is about 7.8% of total plasma T4.

[h] Mendel et al. 1990 reported a value of 1.3 S^{-1}. In the other two studies (Mendel et al. 1988, Mendel and Weisiger 1990), the value was estimated to be > 0.5 S^{-1}.

[i] The review papers by Richardson 2007 and McLean et al. 2017 provided a value of 2174 pM, the review paper by Janssen and Janssen 2017 provided a value of 1000 pM. Murata et al. 1985 reported 704 and 781 pM for Caucasians and Australian aborigines respectively. The Murata paper also summarized a number of studies reporting values ranging from 1282 to 4347 pM. In our model, Kd_{T3TBG} value is set to 1100 pM such that *T3TBG* is about 75% of total plasma T3.

[j] The value 0.165 S^{-1} reported by Hillier 1975 is used in our model. Mendel et al. 1988b reported a value of 0.1 S^{-1} but because of the relatively longer sampling interval used, compared with the Hillier study, the authors estimated that the actual value could be higher than 0.1 S^{-1}.

[k] Chang et al. 1999 and Prapunpoj et al. 2006 reported values of 5.66E4 and 5.326E4 pM respectively. 7.1429E4 and 1.0E6 pM were provided in the review papers by Janssen and Janssen 2017 and McLean et al. 2017 respectively. In our model Kd_{T3TTR} is set to 3.25E5 pM such that *T3TTR* is close to 5% of total plasma T3.

[l] The half-life of T3TTR dissociation is > 1.5 S according to Mendel 1989, which suggests that k_{10} > ln(2)/1.5 = 0.462 S^{-1}. In our model we use the value of 0.69 S^{-1} which was provided in the review paper by Richardson 2007 and suggested by Mendel et al. 1988b.

[m] Yabu et al. 1987 reported a value of 1.6129E6 pM. 5.0E6 and 1.0E7 pM were provided in the review papers by Janssen and Janssen 2017 and

Richardson 2007 respectively. In our model Kd_{T3ALB} is set to 9.75E6 pM such that T3ALB is about 20% of total plasma T3.

[n] The value 2.2 S^{-1} reported by Mendel et al. 1990 is used in our model. Hillier 1975 also reported that the first phase of T3 dissociation from plasma THBPs was finished in < 1 second, which was presumably due to dissociation of T3 from ALB primarily given that T3ALB is more abundant than *T3TTR*. Assuming the half-life of this dissociation is 0.5 second, then ln(2)/0.5 = 1.386 S^{-1} gives a lower limit for k_{12}.

[o] Pilo et al. 1990 reported that the thyroid gland secretes T4 at a rate of 56.2 μg/day/m^2 body surface area (BSA) in. For an average man of 75 kg BW and 1.75 m height, the BSA is about 1.91 m^2, and the corresponding T4 production rate is 56.2*1.91=107 μg/day, which is in line with the value of 102 μg/day for an individual of 70 kg BW reported in Chopra 1976. Our model uses 107 μg/day for an average 75 kg individual. Given that the molecular weight of T4 is 776.87, 107 μg/day is equivalent to 1.6 pmol/S.

[p] k_{21} and k_{28} were estimated together as follows according to flux conservation:

$$k_{21} * fT4 = k_{28} * AT4RBT / VRBT * fu_{T4RBT} + (k_{24}+k_{32}) * AT4RBT * fu_{T4RBT},$$

where *AT4RBT* is the amount of T4 in *RB Tissue*. Therefore,

$$k_{28} * fu_{T4RBT} = (k_{21} * fT4 - (k_{24}+k_{32}) * AT4RBT * fu_{T4RBT}) / (AT4RBT / VRBT),$$

$$k_{28} * fu_{T4RBT} = (k_{21} * 15 - 1.7084E\text{-}6 / fu_{T4RBT} * 6.0874E5 * fu_{T4RBT}) / (6.0874E5 / 61.63),$$

$$k_{28} * fu_{T4RBT} = (k_{21}*15 - 1.04) / 9.877E3.$$

So there is a lower bound for k_{21}, i.e., $k_{21}>0.0693$, to prevent a negative value for k_{28}.

We next took advantage of the four distinct phases of T4 decay in a tracer experiment as described in the Results section in the main text to estimate the k_{21} and k_{28} values, where the distribution of T4 into the *RB Tissue* is responsible for the 3rd phase of the decay.

(i) We start k_{21} with a high value by setting $k_{21} = k_{25}$, i.e., the same influx rate constant as in the *Liver*. We then clamp the free T4 concentration in *RB Blood* at 15 pM, and adjust k_{28} such that the amount of T4 in *RB Tissue, AT4RBT* = 6.0874E5 pmol as indicated in Table S3.

(ii) Next, we run the T4 tracer experiment and examine whether the 3rd decay phase is finished in about one day, according to Pilo et al. 1990 and Sterling and Chodos 1956.

(iii) If not, reduce k_{21} and iterate through steps (i)-(ii) until the 3rd decay phase is finished in about one day. Observe the lower bound of k_{21} set above.

(iv) When k_{21} =1 and k_{28}* fu_{T4RBT} = 0.00142, the 3rd decay phase is finished in about one day. Fine tune k_{28} to 0.00141 to make sure that free T4 in *Body Blood* is 15 pM, and amounts of T4 in *RB Tissue* and T4 in *Liver Tissue* satisfy the percentage values relative to the amount of T4 in the whole body.

[q] It was reported that the thyroid gland secretes T4 and T3 at a rate of 56.2 and 3.34 μg/day/m^2 body surface area (BSA) respectively in Pilo et al. 1990. After adjusting for molecule weight, the molar ratio of T4:T3 thyroid production is (56.2/776.87)/(3.34/651) = 14.1. We decided to use 14 as the production ratio, and as result $k_{22} = k_{20}/14$ = 1.6/14 = 0.1143 pmol/S, which is equivalent 6.43 μg/day for an average man of 75 kg BW, 1.75 m

height, and BSA of 1.91 m^2.

[r] k_{23} and k_{29} were estimated together as follows according to flux conservation:

$k_{23} * fT3 + k_{24} * AT4RBT * fu_{T4RBT} = k_{29} * AT3RBT / VRBT * fu_{T3RBT} + k_{33} * AT3RBT * fu_{T3RBT}$,

$k_{29} * fu_{T3RBT} = (k_{23} * fT3 + k_{24} * AT4RBT * fu_{T4RBT} - k_{33} * AT3RBT * fu_{T3RBT}) / (AT3RBT / VRBT)$.

$k_{29} * fu_{T3RBT} = (k_{23} * 5 - 1.7084e\text{-}6 / fu_{T4RBT} * 0.25 * 6.0874E5 * fu_{T4RBT} - 6.3349E\text{-}6 / fu_{T3RBT} * 4.871E4 * fu_{T3RBT}) / (4.871E4 / 61.63)$,

$k_{29} * fu_{T3RBT} = (k_{23} * 5 - 0.0486) / 790.361$.

So there is a lower bound for k_{23}, i.e., $k_{23} > 0.00972$, to prevent a negative value for k_{29}.

We next took advantage of the four distinct phases of T3 decay in a tracer experiment as described in the Results section in the main text to estimate the k_{23} and k_{29} values, where the distribution of T3 into the *RB Tissue* is responsible for the 3rd phase of the decay.

(i) We start k_{23} with a high value by setting $k_{23} = k_{27}$, i.e., the same influx rate constant as in the *Liver*. We then clamp the free T3 concentration in *RB Blood* at 5 pM, and adjust k_{29} such that the amount of T3 in *RB Tissue, AT3RBT* = 4.871E4 pmol as indicated in Table S3.

(ii) Next, we run the T3 tracer experiment and examine whether the 3rd decay phase is finished in 12-13 h, according to Chopra 1976 and Pilo et al. 1990.

(iii) If not, reduce k_{23} and iterate through steps (i)-(ii) until the 3rd decay phase is finished in about 12-13 h. Observe the lower bound of k_{23} set above.

(iv) When $k_{23} = 0.15$ and $k_{29} * fu_{T4RBT} = 8.8686E\text{-}4$ the 3rd decay phase is finished in about 12-13 h. Fine tune k_{29} to 8.9E-4 to make sure that free T3 in *Body Blood* is 5 pM, and amounts of T3 in *RB Tissue* and T3 in *Liver Tissue* satisfy the percentage values relative to the amount of T3 in the whole body.

[s] k_{24} and k_{32} were estimated together as follows:

$MT4RBTC = 1 - MT4LTC = 1 - 0.35 = 0.65$,

$k_{20} * MT4RBTC = (k_{24}+k_{32}) * AT4RBT * fu_{T4RBT}$,

where *MT4RBTC* is the fraction of T4 metabolism accounted for by the *RB Tissue*. At steady state, T4 metabolism is equal to T4 production (k_{20}). Therefore,

$k_{24} + k_{32} = k_{20} * MT4RBTC / AT4RBT / fu_{T4RBT} = 1.6 * 0.65 / 6.0874E5 / fu_{T4RBT} = 1.7084E\text{-}6 / fu_{T4RBT}\ S^{-1}$.

$k_{24} = a_2 * 1.7084E\text{-}6 / fu_{T4RBT}\ S^{-1}$,

$k_{32} = (1-a_2) * 1.7084E\text{-}6 / fu_{T4RBT}\ S^{-1}$,

where a_2 is the fraction of T4 metabolism in *RB Tissue* accounted for by T4→T3 conversion.

[t] k_{25} was estimated as follows:

$$IT4LC = k_{25} * fT4 / (QL * tT4),$$

where *IT4LC* is the percentage of T4 liver blood supply rate as unidirectional liver uptake rate (influx), *fT4* is the free T4 concentration in *Liver Blood* which is assumed to be the same as the targeted free T4 in the plasma of arterial blood since it only marginally decreases going through the liver, *QL* is the liver plasma flow rate, and *tT4* is the total T4 concentration in the plasma of arterial blood. Therefore,

$$k_{25} = IT4LC / fT4 * QL * tT4 = 0.048 / 15 * 0.0142 * 9.3371E4 = 4.243 \text{ L/S}$$

Equivalently, k_{25} can also be obtained by clamping *fT4* in all blood compartments at 15 pM then adjusting k_{25} until *IT4LC* = 0.048.

[u] k_{26} and k_{34} were estimated together as follows:

$$k_{20} * MT4LTC = (k_{26}+k_{34}) * AT4LT * fu_{T4LT},$$

where *AT4LT* is the amount of T4 in *Liver Tissue*, *AT4WB* is the extrathyroidal amount of T4 in the whole body, and *AT4LTC* is the *Liver Tissue* fraction of *AT4WB*, *MT4LTC* is the fraction of T4 metabolism accounted for by the *Liver Tissue*, and fu_{T4LT} is free T4 fraction in *Liver Tissue*. At steady state, the overall T4 metabolism is equal to T4 production (k_{20}). Therefore,

$$k_{26}+ k_{34} = k_{20} * MT4LTC / AT4LT / fu_{T4LT} = 1.6 * 0.35 / 3.8889E5 / fu_{T4LT} = 1.44E\text{-}6 / fu_{T4LT} \text{ S}^{-1},$$

$$k_{26} = a_1 * 1.44E\text{-}6 / fu_{T4LT} \text{ S}^{-1},$$

$$k_{34} = (1\text{-}a_1) * 1.44E\text{-}6 / fu_{T4LT} \text{ S}^{-1},$$

where a_1 is the fraction of T4 metabolism in *Liver Tissue* accounted for by T4→T3 conversion.

[v] k_{27} was estimated as follows:

$$IT3LC = k_{27} * fT3 / (QL * tT3),$$

where *IT3LC* is the percentage of T3 liver blood supply rate as unidirectional liver uptake rate (influx), *fT3* is the free T3 concentration in *Liver Blood* which is assumed to be the same as the targeted free T3 in the plasma of arterial blood since it only marginally decreases going through the liver, *QL* is the liver plasma flow rate, and *tT3* is the total T3 concentration in the plasma of arterial blood (which is estimated by clamping *fT3* to 5 pM and measuring total T3 concentration in the Body Blood compartment.). Therefore,

$$k_{27} = IT3LC / fT3 * QL * tT3 = 0.29 / 5 * 0.0142 * 1.698E3 = 1.3985 \text{ L/S}.$$

Equivalently, k_{27} can also be obtained by clamping *fT3* in all blood compartments at 5 pM then adjusting k_{27} until *IT3LC* = 0.29.

[w] See calculation for k_{21}.

[x] See calculation for k_{23}.

[y] With the liver-related T4 parameters k_{25}, k_{26}, and k_{34} determined, k_{30} was estimated as follows:

Clamping *fT4* in the plasma of *Live Blood* at 15 pM, then adjust k_{30} until *AT4LT* = 3.8889E5 pmol as indicated in Table S3, which is 30% (*AT4LTC*) of *AT4WB*. As a result, k_{30} = 2.786-4/fu_{T4LT} L/S.

[z] With the liver-related T3 parameters k_{26}, k_{27}, and k_{35} determined, k_{31} was estimated as follows:

Clamping *fT3* in the plasma of *Live Blood* at 5 pM, then adjust k_{31} until *AT3LT* = 6.0099E3 pmol as indicated in Table S3, which is 10% (*AT3LTC*) of *AT3WB*. As a result, k_{31} = 1.9688E-3/fu_{T3LT} L/S.

[aa] See calculation for k_{24}.

[bb] k_{33} was estimated as follows:

$$MT3RBTC = 1 - MT3LTC = 1 - 0.4 = 0.6,$$

$$(k_{22} + k_{20}*a_2) * MT3RBTC = k_{33} * AT3RBT * fu_{T3RBT},$$

where *MT3RBTC* is the fraction of T3 metabolism accounted for by the *RB Tissue*. At steady state, the overall T3 metabolism is equal to T3 production ($k_{22} + k_{20}*a_2$ or $k_{22} + k_{20}*a_1$, where a_1=a_2). Therefore,

$$k_{33} = (k_{22} + k_{20}*a_2) * MT3RBTC / AT3RBT / fu_{T3RBT} = (0.1143 + 1.6*0.25) * 0.6 /4.871E4 / fu_{T3RBT},$$

$$k_{33} = 6.3349E\text{-}6 / fu_{T3RBT}\ S^{-1}.$$

[cc] See calculation for k_{26}.

[dd] k_{35} was estimated as follows:

$$MT3LTC * (k_{22} + k_{20} * a_1) = k_{35} * AT3LT * fu_{T3LT},$$

where ($k_{22} + k_{20} * a_1$) is the total production rate of T3. Therefore,

$$k_{35} = MT3LTC * (k_{22} + k_{20} * a_1) / AT3LT / fu_{T3LT},$$

$$k_{35} = 0.4 * (1.6/14 + 1.6*0.25) / 6.0099E3 / fu_{T3LT} = 3.4229E\text{-}5/fu_{T3LT}\ S^{-1}.$$

[ee] Pilo et al. 1990 estimated a range of 25.6%-27.3% for the percentage of peripheral T4 converted to T3. Maia et al. 2005 estimated that T3 productions by liver and muscle add up to 44 nmol/day when plasma free T4 is 20 pM for 70-kg human. Assuming a linear relationship, then scaling for the condition of plasma free T4 at 15 pM and 75-kg human, as in our model, the T3 production will be 44*(15/20)*(75/70)/24/3600*1000=0.4092 pMol/S. Since the T4 production is 1.6 pMol/S in our model, then the values provide a value of 0.4092/1.6 = 25.57% for the percentage of peripheral T4 converted to T3. Lastly, Chopra 1976 reported total T4 production is 102 μg/day/70 kg, total T3 production is 32.4 μg/day/70kg, and 76.2% of which comes from T4. Considering the molecular weights of T3 (651) and T4 (776.87), these values provide a percentage of (32.4*0.762/651)/(102/776.87) = 28.88%. We use the value of 0.25 for both a_1 and a_2 assuming similar percentage conversion of T4 to T3 in both the *Liver* and *RB* compartments.

[ff] The actually values of these free fraction are not important in the model because the parameters k_{24}, k_{26}, k_{28}-k_{35} are all scaled by these Fu constants, so they cancel out in the rate equations. In their modeling paper, Pardridge and Landaw (1987) estimated that free and bound T3 are 0.53 and 4.2 nM in the cytosol of human liver. This gave an estimate of 0.53/(0.53+4.2)=0.11 as the free fraction of T3 in the liver.

[gg] Total plasma concentration of human TBG has been reported from multiple sources. The values reported in early studies and cited in a number of review articles tend to be low compared with the reference ranges measured in multiple modern laboratories. Richardson 2007, McLean et al. 2017, and Janssen and Janssen 2017, and Franklyn and Shepard 2000 cited mean values between 15-16 µg/ml. Attwood and Atkin 1982 cited a range of 6.1-16 µg/ml, Franklyn et al. cited a range of 11-22 µg/ml, and Attwood et al. 1978 reported a range of 8-15 µg/ml. In comparison, multiple modern laboratories reported higher reference ranges, such as 12-26 (M), 11-27 (F) µg/ml from Mayo Clinic, 12.7-25.1 (M), 13.5-30.9 (F) µg/ml for adults from QuestDiagnostics, 13-39 (M and F) µg/ml for adults from LabCorp, and 12-26 (M) and 11-27 (F) µg/ml from study.com. The model used 3.515E5 pM which is about 19 µg/ml.

[hh] Total plasma concentration of human TTR homotetramer has been reported from multiple sources. Janssen and Janssen 2017, McLean et al. 2017, and Richardson 2007 cited 250 mg/L, Franklyn cited a range of 160-300 mg/L, and Schussler 2000 cited 4.6E6 pM. Vataserry 1991 reported a range of 170-420 mg/L. The model used 5.35E6 pM which is about 294 mg/L.

[ii] Total plasma concentration of human albumin has been reported from multiple sources. McLean et al. 2017 and Richardson 2007 cited 42000 mg/L, Janssen and Janssen 2017 cited 40000 mg/L, Schussler 2000 cited 6.4E8 pM. Barlow et al. 1982 reported 6.0E8 pM, and Gardner and Scott 1980 reported a range of 6.89E8-8.27E8 pM. The model used 6.45E8 pM which is about 42893 mg/L.

[jj] Mendel 1989, Richardson 2007, and McLean et al. 2017 cited 0.03% as the free T4 fraction in plasma. Schussler 2000 cited 0.02%. A range of 0.005%-0.03% was cited on EndocrineWeb.

[kk] The reported values and ranges vary from laboratories and with the methods used. For instance, Jain 2015 reported a geomean of 0.8 ng/dL (10.3 pM) based on the 2007-2012 US NHANES survey. Franklyn and Shepard 2000 cited a range of 13-39 pM. Mayo Clinic uses 0.9-1.7 ng/dl (11.58-221.88 pM) as the reference range for adults, GlobalRPh uses (10-23 pM), and EndocrineWeb reports 0.7-1.9 ng/dl (9.01-24.46 pM) as the range. The model uses 15 pM which is within these ranges and, more importantly, it allows the model to produce steady-state results that are consistent with values reported in the literature for plasma distributions, such as percentage of free T4, total T4, percentage T4TBG, T4TTR and T4ALB, and percentage saturation of TBG, TTR and ALB.

[ll] The reported values and ranges vary from laboratories and with the methods used. For instance, Jain 2015 reported a geomean of 3.22 pg/mL (4.96 pM) based on the 2007-2012 US NHANES survey. Franklyn and Shepard 2000 cited a range of 3.8-10 pM. Mayo Clinic uses 280-440 pg/dl (4.3-6.76 pM) as the reference range for adults, GlobalRPh uses 3.5-6.5 pM, and EndocrineWeb reports 230-619 pg/dl (3.53-9.51 pM) as the range. The model uses 5 pM which is within these ranges and, more importantly, it allows the model to produce steady-state results that are consistent with values reported in the literature for plasma distributions, such as percentage of free T3, total T3, percentage T3TBG, T3TTR and T3ALB.

[mm] The reported values and ranges vary. Aoki et al. 2007 reported 9.79±0.9*E4 (mean±SE) pM for US population not taking thyroid medication in the 1999-2002 cycles of the NHANES survey. Jain 2015 reported a geomean of 7.47 µg/dl (9.615E4 pM) in the 2007-2012 cycles of the NHANES

survey. Franklyn and Shepard 2000 cited a range of 6.4E4-15.4E4 pM. Mayo Clinic uses 4.5-11.7 µg/dl (5.79E4-15.06E4 pM) as the reference range for adults, GlobalRPh uses 5.8E4-16.1E4 nM, and EndocrineWeb reports 4.6-12 µg/dl (5.92E4-15.45E4 pM) as the range. The total T4 in our model is at 9.3371E4 pM, which is close to the means above and in the ranges cited here.

[nn] The reported values and ranges vary. Jain 2015 reported a geomean of 115.38 ng/dl (1.772E3 pM) in the 2007-2012 cycles of the NHANES survey. Franklyn and Shepard 2000 cited a range of 1.2E3-2.9E3 pM. Mayo Clinic uses 80-200 ng/dl (1.23E3-3.08E3 pM) as the reference range for adults, GlobalRPh uses 80-200 ng/dl (1.23E3-3.08E3 pM), and EndocrineWeb reports 80-180 ng/dl (1.23E3-2.77E3 pM) as the range. The total T3 in our model is at 1694 pM, which is close to the mean and in the ranges cited here.

[oo] In their modeling paper, Pardridge and Landaw (1987) estimated that free and bound T3 are 0.53 and 4.2 nM in the cytosol of human liver. It was also estimated in the paper that the free nuclear T3 in human liver is about 1 nM based on the assumption that TR (which has a *Kd* of 1 nM) is 50% saturated by T3.

[pp] Based on the study by Cavalieri and Searle 1966, the T4 hepatic distribution volume is 3.8 L and its total distribution volume is 12.4 L. Therefore, the percentage of extrathyroidal T4 in the liver can be estimated as 3.8/12.4 = 30.65%. In the paper by Felicetta et al. 1980, a range of 14%-30% were estimated based on many studies, but 30% is favored and supported by the majority of the cited studies. Therefore, 30% is used in our model.

[qq] Using a 5-pool model, Curti and Fresco 1992 estimated that liver T3 is about 10% of the extrathyroidal T3. In a multi-compartment kinetic analysis, Pilo et al. 1990 estimated that T3 in the fast pool which includes liver is 12.9% of the extrathyroidal T3. Cavalieri et al. 1970 estimated that liver T3 is about 5% of the extrathyroidal T3. 10% is used in our model.

[rr] Maia et al. 2005 estimated that in a 70-kg euthyroid human, at 20 pM of free T4 level, the hepatic DIO1-catalyzed T4 to T3 conversion produces about 15 nmol of T3/day. Using linear scaling for a 75-kg human with 15 pM free T4 level, the liver production rate of T3 is 15*(75/70)*(15/20) = 12 nmol/day.

[ss] Maia et al. 2005 estimated that in a 70-kg euthyroid human, at 20 pM of free T4 level, the muscular DIO2-catalyzed T4 to T3 conversion produces about 29 nmol of T3/day. Using linear scaling for a 75-kg human with 15 pM free T4 level, the muscular production rate of T3 is 29*(75/70)*(15/20) = 23.3 nmol/day.

[tt] Nicoloff et al. 1972 reported a total mass production ratio of T3/T4 as 27.6/88.5 = 0.31, which can be converted to molar production ratio according to (27.6/651)/(88.5/776.87) = 0.37. Chopra 1976 reported for a 70-kg human a total mass production ratio of T3/T4 as 32.4/102=0.32, which can be converted to molar production ratio according to (32.4/651)/(102/776.87) = 0.38.

[uu] Both Sterling and Chodos 1956 and Cavalieri and Searle 1966 reported a half-life of T4 of 6.7 days in euthyroid men. Nicoloff 1972 reported a T4 turnover rate of 0.112/day in in euthyroid men, which is equivalent to a half-life of log(2)/0.112 = 6.2 days. We used the average of 6.5 days as our targeted half-life of extrathyroidal T4 for parameter estimation.

[vv] Cavalieri et al. 1971 reported a half-life of T3 of 22.3 hours in euthyroid men. Nicoloff et al. 1972 reported a T3 turnover rate of 0.679/day in euthyroid men, which is equivalent to a half-life of log(2)/0.679*24 = 24.5 hours. Jonklaas 2015 reported a half-life of T3 of 22 hours in euthyroid men with orally administrated LT3. The average of these numbers are 22.9 hours. We used 22.5 hours as our targeted half-life of extrathyroidal

T3 for parameter estimation.

[ww] In the paper by Mendel et al. 1988, 35% of T4 was assumed to be metabolized in the liver in humans.

[xx] In the review article by Mendel 1989, four values were provided in Table 3 citing various studies. We take the mean of these values, which is 4.8%.

[yy] Using a 5-pool model, Curti and Fresco 1992 estimated that liver can be responsible for 41% of T3 metabolism if including those eliminated through the bile and eventually through the feces. 40% is used in our model.

References

Aoki, Y., R. M. Belin, R. Clickner, R. Jeffries, L. Phillips and K. R. Mahaffey (2007). "Serum TSH and total T4 in the United States population and their association with participant characteristics: National Health and Nutrition Examination Survey (NHANES 1999-2002)." Thyroid **17**(12): 1211-1223.

Attwood, E. C. and G. E. Atkin (1982). "The T4:TBG ratio: a re-evaluation with particular reference to low and high serum TBG levels." Ann Clin Biochem **19**(Pt 2): 101-103.

Attwood, E. C., R. M. Seddon and D. E. Probert (1978). "The T4/TBG ratio and the investigation of thyroid function." Clin Biochem **11**(5): 218-221.

Barlow, J. W., J. M. Csicsmann, E. L. White, J. W. Funder and J. R. Stockigt (1982). "Familial euthyroid thyroxine excess: characterization of abnormal intermediate affinity thyroxine binding to albumin." J Clin Endocrinol Metab **55**(2): 244-250.

Brown, R. P., M. D. Delp, S. L. Lindstedt, L. R. Rhomberg and R. P. Beliles (1997). "Physiological parameter values for physiologically based pharmacokinetic models." Toxicol Ind Health **13**(4): 407-484.

Cavalieri, R. R. and G. L. Searle (1966). "The kinetics of distribution between plasma and liver of 131-I-labeled L-thyroxine in man: observations of subjects with normal and decreased serum thyroxine-binding globulin." J Clin Invest **45**(6): 939-949.

Cavalieri, R. R., M. Steinberg and G. L. Searle (1970). "The distribution kinetics of triiodothyronine: studies of euthyroid subjects with decreased plasma thyroxine-binding globulin and patients with Graves' disease." J Clin Invest **49**(6): 1041-1050.

Cavalieri, R. R., M. Steinberg and G. L. Searle (1971). "Metabolic clearance rate of L-triiodothyronine in man: a comparison of results by single-injection and constant infusion methods." J Clin Endocrinol Metab **33**(4): 624-629.

Chang, L., S. L. Munro, S. J. Richardson and G. Schreiber (1999). "Evolution of thyroid hormone binding by transthyretins in birds and mammals." Eur J Biochem **259**(1-2): 534-542.

Chopra, I. J. (1976). "An assessment of daily production and significance of thyroidal secretion of 3, 3', 5'-triiodothyronine (reverse T3) in man." J Clin Invest **58**(1): 32-40.

Curti, G. L. and G. F. Fresco (1992). "A theoretical five-pool model to evaluate triiodothyronine distribution and metabolism in healthy subjects." Metabolism **41**(1): 3-10.

Davies, B. and T. Morris (1993). "Physiological parameters in laboratory animals and humans." Pharm Res **10**(7): 1093-1095.

Felicetta, J. V., W. L. Green and W. B. Nelp (1980). "Inhibition of hepatic binding of thyroxine by cholecystographic agents." J Clin Invest **65**(5): 1032-1040.

Franklyn, J. and M. Shepard (2000). Evaluation of Thyroid Function in Health and Disease. Endotext. K. R. Feingold, B. Anawalt, A. Boyce et al. South Dartmouth (MA), MDText.com, Inc.

Copyright © 2000-2021, MDText.com, Inc.

Gardner, M. D. and R. Scott (1980). "Age- and sex-related reference ranges for eight plasma constituents derived from randomly selected adults in a Scottish new town." J Clin Pathol **33**(4): 380-385.

Hillier, A. P. (1971). "Human thyroxine-binding globulin and thyroxine-binding pre-albumin: dissociation rates." J Physiol **217**(3): 625-634.

Hillier, A. P. (1975). "The rate of triiodothyronine dissociation from binding sites in human plasma." Acta Endocrinol (Copenh) **80**(1): 49-57.

Jain, R. (2015). "Thyroid profile of the reference United States population: Data from NHANES 2007-2012." Int Arch Endocrinol Clin Res **1**(1): 1-8.

Janssen, S. T. and O. E. Janssen (2017). "Directional thyroid hormone distribution via the blood stream to target sites." Molecular and Cellular Endocrinology **458**: 16-21.

Jonklaas, J., K. D. Burman, H. Wang and K. R. Latham (2015). "Single-dose T3 administration: kinetics and effects on biochemical and physiological parameters." Ther Drug Monit **37**(1): 110-118.

Maia, A. L., B. W. Kim, S. A. Huang, J. W. Harney and P. R. Larsen (2005). "Type 2 iodothyronine deiodinase is the major source of plasma T3 in euthyroid humans." J Clin Invest **115**(9): 2524-2533.

McLean, T. R., M. M. Rank, P. M. Smooker and S. J. Richardson (2017). "Evolution of thyroid hormone distributor proteins." Mol Cell Endocrinol **459**: 43-52.

Mendel, C. M. (1989). "The free hormone hypothesis: a physiologically based mathematical model." Endocr Rev **10**(3): 232-274.

Mendel, C. M., R. R. Cavalieri and R. A. Weisiger (1988a). "Uptake of thyroxine by the perfused rat liver: implications for the free hormone hypothesis." Am J Physiol **255**(2 Pt 1): E110-119.

Mendel, C. M., M. B. Miller, P. K. Siiteri and J. T. Murai (1990). "Rates of dissociation of steroid and thyroid hormones from human serum albumin." J Steroid Biochem Mol Biol **37**(2): 245-250.

Mendel, C. M. and R. A. Weisiger (1990). "Thyroxine uptake by perfused rat liver. No evidence for facilitation by five different thyroxine-binding proteins." J Clin Invest **86**(6): 1840-1847.

Mendel, C. M., R. A. Weisiger and R. R. Cavalieri (1988b). "Uptake of 3,5,3'-triiodothyronine by the perfused rat liver: return to the free hormone hypothesis." Endocrinology **123**(4): 1817-1824.

Murata, Y., S. Refetoff, D. H. Sarne, M. Dick and F. Watson (1985). "Variant thyroxine-binding globulin in serum of Australian aborigines: its physical, chemical and biological properties." J Endocrinol Invest **8**(3): 225-232.

Nicoloff, J. T., J. C. Low, J. H. Dussault and D. A. Fisher (1972). "Simultaneous measurement of thyroxine and triiodothyronine peripheral turnover kinetics in man." J Clin Invest **51**(3): 473-483.

Pardridge, W. M. and E. M. Landaw (1987). "Steady state model of 3,5,3'-triiodothyronine transport in liver predicts high cellular exchangeable hormone concentration relative to in vitro free hormone concentration." Endocrinology **120**(3): 1059-1068.

Petitpas, I., C. E. Petersen, C. E. Ha, A. A. Bhattacharya, P. A. Zunszain, J. Ghuman, N. V. Bhagavan and S. Curry (2003). "Structural basis of albumin-thyroxine interactions and familial dysalbuminemic hyperthyroxinemia." Proc Natl Acad Sci U S A **100**(11): 6440-6445.

Pilo, A., G. Iervasi, F. Vitek, M. Ferdeghini, F. Cazzuola and R. Bianchi (1990). "Thyroidal and peripheral production of 3,5,3'-triiodothyronine in humans by multicompartmental analysis." Am J Physiol **258**(4 Pt 1): E715-726.

Poulin, P. and F. P. Theil (2002). "Prediction of pharmacokinetics prior to in vivo studies. 1. Mechanism-based prediction of volume of distribution." J Pharm Sci **91**(1): 129-156.

Prapunpoj, P., L. Leelawatwatana, G. Schreiber and S. J. Richardson (2006). "Change in structure of the N-terminal region of transthyretin produces change in affinity of transthyretin to T4 and T3." Febs j **273**(17): 4013-4023.

Richardson, S. J. (2007). "Cell and molecular biology of transthyretin and thyroid hormones." Int Rev Cytol **258**: 137-193.

Schussler, G. C. (2000). "The thyroxine-binding proteins." Thyroid **10**(2): 141-149.

Sterling, K. and R. B. Chodos (1956). "Radiothyroxine turnover studies in myxedema, thyrotoxicosis, and hypermetabolism without endocrine disease." J Clin Invest **35**(7): 806-813.

Vatassery, G. T., H. T. Quach, W. E. Smith, B. A. Benson and J. H. Eckfeldt (1991). "A sensitive assay of transthyretin (prealbumin) in human cerebrospinal fluid in nanogram amounts by ELISA." Clin Chim Acta **197**(1): 19-25.

Yabu, Y., K. Miyai, A. Kobayashi, K. Miki, K. Doi, J. Takamatsu, T. Mozai, F. Matsuzuka and K. Kuma (1987). "A new type of albumin with predominantly increased binding affinity for 3,3',5-triiodothyronine in a patient with Graves' disease." J Endocrinol Invest **10**(2): 163-169.
