# Supplementary material for: A minimal human physiologically based kinetic model of thyroid hormones and chemical disruption of plasma thyroid hormone binding proteins
Source: Front Endocrinol (Lausanne). 2023 May 25;14:1168663. doi: 10.3389/fendo.2023.1168663 (PMC10248451; doi:10.3389/fendo.2023.1168663)
Supplement: Supplementary file 3 [file Table_2.pdf]

Table S4. ODEs and algebraic equations of the nonspatial PBK Model

State Variable			ODE
Free T4 in *Body Blood*:	fT4B'	=	-k1*fT4B*TBGB + k2*T4TBGB - k3*fT4B*TTRB + k4*T4TTRB - k5*fT4B*ALBB + k6*T4ALBB + (fT4T*QT + fT4RB*QRB + fT4L*QL - fT4B*QC)/VB
T4TBG in *Body Blood*:	T4TBGB'	=	k1*fT4B*TBGB - k2*T4TBGB + (T4TBGT*QT + T4TBGRB*QRB + T4TBGL*QL - T4TBGB*QC)/VB
T4TTR in *Body Blood*:	T4TTRB'	=	k3*fT4B*TTRB - k4*T4TTRB + (T4TTRT*QT + T4TTRRB*QRB + T4TTRL*QL - T4TTRB*QC)/VB
T4ALB in *Body Blood*:	T4ALBB'	=	k5*fT4B*ALBB - k6*T4ALBB + (T4ALBT*QT + T4ALBRB*QRB + T4ALBL*QL - T4ALBB*QC)/VB
Free T3 in *Body Blood*:	fT3B'	=	-k7*fT3B*TBGB + k8*T3TBGB - k9*fT3B*TTRB + k10*T3TTRB - k11*fT3B*ALBB + k12*T3ALBB + (fT3T*QT + fT3RB*QRB + fT3L*QL - fT3B*QC)/VB
T3TBG in *Body Blood*:	T3TBGB'	=	k7*fT3B*TBGB - k8*T3TBGB + (T3TBGT*QT + T3TBGRB*QRB + T3TBGL*QL - T3TBGB*QC)/VB
T3TTR in *Body Blood*:	T3TTRB'	=	k9*fT3B*TTRB - k10*T3TTRB + (T3TTRT*QT + T3TTRRB*QRB + T3TTRL*QL - T3TTRB*QC)/VB
T3ALB in *Body Blood*:	T3ALBB'	=	k11*fT3B*ALBB - k12*T3ALBB + (T3ALBT*QT + T3ALBRB*QRB + T3ALBL*QL - T3ALBB*QC)/VB
Free TBG in *Body Blood*:	TBGB'	=	-k1*fT4B*TBGB + k2*T4TBGB - k7*fT3B*TBGB + k8*T3TBGB - k42*XB*TBGB + k43*XTBGB + (TBGT*QT + TBGRB*QRB + TBGL*QL - TBGB*QC)/VB
Free TTR in *Body Blood*:	TTRB'	=	-k3*fT4B*TTRB + k4*T4TTRB - k9*fT3B*TTRB + k10*T3TTRB - k38*XB*TTRB + k39*XTTRB + (TTRT*QT + TTRRB*QRB + TTRL*QL - TTRB*QC)/VB
Free ALB in *Body Blood*:	ALBB'	=	-k5*fT4B*ALBB + k6*T4ALBB - k11*fT3B*ALBB + k12*T3ALBB + (ALBT*QT + ALBRB*QRB + ALBL*QL - ALBB*QC)/VB;
Free T4 in *Thyroid Blood*:	fT4T'	=	-k1*fT4T*TBGT + k2*T4TBGT - k3*fT4T*TTRT + k4*T4TTRT - k5*fT4T*ALBT + k6*T4ALBT + k20/VTB + (fT4B-fT4T)*QT/VTB
T4TBG in *Thyroid blood*:	T4TBGT'	=	k1*fT4T*TBGT - k2*T4TBGT + (T4TBGB-T4TBGT)*QT/VTB
T4TTR in *Thyroid blood*:	T4TTRT'	=	k3*fT4T*TTRT - k4*T4TTRT + (T4TTRB-T4TTRT)*QT/VTB
T4ALB in *Thyroid blood*:	T4ALBT'	=	k5*fT4T*ALBT - k6*T4ALBT + (T4ALBB-T4ALBT)*QT/VTB
Free T3 in *Thyroid blood*:	fT3T'	=	-k7*fT3T*TBGT + k8*T3TBGT - k9*fT3T*TTRT + k10*T3TTRT - k11*fT3T*ALBT + k12*T3ALBT + k22/VTB + (fT3B-fT3T)*QT/VTB
T3TBG in *Thyroid blood*:	T3TBGT'	=	k7*fT3T*TBGT - k8*T3TBGT + (T3TBGB-T3TBGT)*QT/VTB
T3TTR in *Thyroid blood*:	T3TTRT'	=	k9*fT3T*TTRT - k10*T3TTRT + (T3TTRB-T3TTRT)*QT/VTB
T3ALB in *Thyroid blood*:	T3ALBT'	=	k11*fT3T*ALBT - k12*T3ALBT + (T3ALBB-T3ALBT)*QT/VTB
Free TBG in *Thyroid blood*:	TBGT'	=	-k1*fT4T*TBGT + k2*T4TBGT - k7*fT3T*TBGT + k8*T3TBGT - k42*XT*TBGT + k43*XTBGT + (TBGB-TBGT)*QT/VTB

Free TTR in *Thyroid blood*:	TTRT'	=	-k3*fT4T*TTRT + k4*T4TTRT - k9*fT3T*TTRT + k10*T3TTRT - k38*XT*TTRT + k39*XTTRT + (TTRB-TTRT)*QT/VTB
Free ALB in *Thyroid blood*:	ALBT'	=	-k5*fT4T*ALBT + k6*T4ALBT - k11*fT3T*ALBT + k12*T3ALBT + (ALBB-ALBT)*QT/VTB
Free T4 in *RB blood*:	fT4RB'	=	-k1*fT4RB*TBGRB + k2*T4TBGRB - k3*fT4RB*TTRRB + k4*T4TTRRB - k5*fT4RB*ALBRB + k6*T4ALBRB + (-k21*fT4RB + k28*T4RBT*fuT4RBT)/VRBB + (fT4B-fT4RB)*QRB/VRBB
T4TBG in *RB blood*:	T4TBGRB'	=	k1*fT4RB*TBGRB - k2*T4TBGRB + (T4TBGB-T4TBGRB)*QRB/VRBB
T4TTR in *RB blood*:	T4TTRRB'	=	k3*fT4RB*TTRRB - k4*T4TTRRB + (T4TTRB-T4TTRRB)*QRB/VRBB
T4ALB in *RB blood*:	T4ALBRB'	=	k5*fT4RB*ALBRB - k6*T4ALBRB + (T4ALBB-T4ALBRB)*QRB/VRBB
Free T3 in *RB blood*:	fT3RB'	=	-k7*fT3RB*TBGRB + k8*T3TBGRB - k9*fT3RB*TTRRB + k10*T3TTRRB - k11*fT3RB*ALBRB + k12*T3ALBRB + (-k23*fT3RB + k29*T3RBT*fuT3RBT)/VRBB + (fT3B-fT3RB)*QRB/VRBB
T3TBG in *RB blood*:	T3TBGRB'	=	k7*fT3RB*TBGRB - k8*T3TBGRB + (T3TBGB-T3TBGRB)*QRB/VRBB
T3TTR in *RB blood*:	T3TTRRB'	=	k9*fT3RB*TTRRB - k10*T3TTRRB + (T3TTRB-T3TTRRB)*QRB/VRBB
T3ALB in *RB blood*:	T3ALBRB'	=	k11*fT3RB*ALBRB - k12*T3ALBRB + (T3ALBB-T3ALBRB)*QRB/VRBB
Free TBG in *RB blood*:	TBGRB'	=	-k1*fT4RB*TBGRB + k2*T4TBGRB - k7*fT3RB*TBGRB + k8*T3TBGRB - k42*XRB*TBGRB + k43*XTBGRB + (TBGB-TBGRB)*QRB/VRBB
Free TTR in *RB blood*:	TTRRB'	=	-k3*fT4RB*TTRRB + k4*T4TTRRB - k9*fT3RB*TTRRB + k10*T3TTRRB - k38*XRB*TTRRB + k39*XTTRRB + (TTRB-TTRRB)*QRB/VRBB
Free ALB in *RB blood*:	ALBRB'	=	-k5*fT4RB*ALBRB + k6*T4ALBRB - k11*fT3RB*ALBRB + k12*T3ALBRB + (ALBB-ALBRB)*QRB/VRBB
T4 in *RB tissue*:	T4RBT'	=	(k21*fT4RB - k28*T4RBT*fuT4RBT)/VRBT - k24*T4RBT*fuT4RBT - k32*T4RBT*fuT4RBT
T3 in *RB tissue*:	T3RBT'	=	(k23*fT3RB - k29*T3RBT*fuT3RBT)/VRBT + k24*T4RBT*fuT4RBT - k33*T3RBT*fuT3RBT
Free T4 in *Liver blood*:	fT4L'	=	-k1*fT4L*TBGL + k2*T4TBGL - k3*fT4L*TTRL + k4*T4TTRL - k5*fT4L*ALBL + k6*T4ALBL + (-k25*fT4L + k30*T4LT*fuT4LT)/VLB + (fT4B-fT4L)*QL/VLB
T4TBG in *Liver blood*:	T4TBGL'	=	k1*fT4L*TBGL - k2*T4TBGL + (T4TBGB-T4TBGL)*QL/VLB
T4TTR in *Liver blood*:	T4TTRL'	=	k3*fT4L*TTRL - k4*T4TTRL + (T4TTRB-T4TTRL)*QL/VLB
T4ALB in *Liver blood*:	T4ALBL'	=	k5*fT4L*ALBL - k6*T4ALBL + (T4ALBB-T4ALBL)*QL/VLB
Free T3 in *Liver blood*:	fT3L'	=	-k7*fT3L*TBGL + k8*T3TBGL - k9*fT3L*TTRL + k10*T3TTRL - k11*fT3L*ALBL + k12*T3ALBL + (-k27*fT3L + k31*T3LT*fuT3LT)/VLB + (fT3B-fT3L)*QL/VLB
T3TBG in *Liver blood*:	T3TBGL'	=	k7*fT3L*TBGL - k8*T3TBGL + (T3TBGB-T3TBGL)*QL/VLB
T3TTR in *Liver blood*:	T3TTRL'	=	k9*fT3L*TTRL - k10*T3TTRL + (T3TTRB-T3TTRL)*QL/VLB
T3ALB in *Liver blood*:	T3ALBL'	=	k11*fT3L*ALBL - k12*T3ALBL + (T3ALBB-T3ALBL)*QL/VLB

T4 in *Liver tissue*:	T4LT'	=	(k25*fT4L - k30*T4LT*fuT4LT)/VLT - k26*T4LT*fuT4LT - k34*T4LT*fuT4LT
T3 in *Liver tissue*:	T3LT'	=	(k27*fT3L - k31*T3LT*fuT3LT)/VLT + k26*T4LT*fuT4LT - k35*T3LT*fuT3LT
Free X in *Body Blood*:	XB'	=	k36/VB - k37*XB - k38*XB*TTRB - k42*XB*TBGB + k39*XTTRB + k43*XTBGB + (XT*QT + XRB*QRB + XL*QL - XB*QC)/VB
XTTR in *Body Blood*:	XTTRB'	=	k38*XB*TTRB - k39*XTTRB + (XTTRT*QT + XTTRRB*QRB + XTTRL*QL - XTTRB*QC)/VB
Free X in *Thyroid blood*:	XT'	=	-k38*XT*TTRT - k42*XT*TBGT + k39*XTTRT + k43*XTBGT + (XB-XT)*QT/VTB
XTTR in *Thyroid blood*:	XTTRT'	=	k38*XT*TTRT - k39*XTTRT + (XTTRB-XTTRT)*QT/VTB
Free X in *RB blood*:	XRB'	=	-k38*XRB*TTRRB - k42*XRB*TBGRB + k39*XTTRRB + k43*XTBGRB + (XB-XRB)*QRB/VRBB
XTTR in *RB blood*:	XTTRRB'	=	k38*XRB*TTRRB - k39*XTTRRB + (XTTRB-XTTRRB)*QRB/VRBB
Free X in *Liver blood*:	XL'	=	-k38*XL*TTRL - k42*XL*TBGL + k39*XTTRL + k43*XTBGL + (XB-XL)*QL/VLB
XTTR in *Liver blood*:	XTTRL'	=	k38*XL*TTRL - k39*XTTRL + (XTTRB-XTTRL)*QL/VLB
XTBG in *Body Blood*:	XTBGB'	=	k42*XB*TBGB - k43*XTBGB + (XTBGT*QT + XTBGRB*QRB + XTBGL*QL - XTBGB*QC)/VB
XTBG in *Thyroid blood*:	XTBGT'	=	k42*XT*TBGT - k43*XTBGT + (XTBGB-XTBGT)*QT/VTB
XTBG in *RB blood*:	XTBGRB'	=	k42*XRB*TBGRB - k43*XTBGRB + (XTBGB-XTBGRB)*QRB/VRBB
XTBG in *Liver blood*:	XTBGL'	=	k42*XL*TBGL - k43*XTBGL + (XTBGB-XTBGL)*QL/VLB
**Derived Variables**			**Algebraic Equation**
Free TBG in *Liver blood*:	TBGL	=	(TBGtot - (TBGB + T4TBGB + T3TBGB + XTBGB)*VB - (TBGT + T4TBGT + T3TBGT + XTBGT)*VTB - (TBGRB + T4TBGRB + T3TBGRB + XTBGRB)*VRBB - (T4TBGL + T3TBGL + XTBGL)*VLB)/VLB
Free TTR in *Liver blood*:	TTRL	=	(TTRtot - (TTRB + T4TTRB + T3TTRB + XTTRB)*VB - (TTRT + T4TTRT + T3TTRT + XTTRT)*VTB - (TTRRB + T4TTRRB + T3TTRRB + XTTRRB)*VRBB - (T4TTRL + T3TTRL + XTTRL)*VLB)/VLB
Free ALB in *Liver blood*:	ALBL	=	(ALBtot - (ALBB + T4ALBB + T3ALBB)*VB - (ALBT + T4ALBT + T3ALBT)*VTB - (ALBRB + T4ALBRB + T3ALBRB)*VRBB - (T4ALBL + T3ALBL)*VLB)/VLB
