# Supplementary material for: A minimal human physiologically based kinetic model of thyroid hormones and chemical disruption of plasma thyroid hormone binding proteins
Source: Front Endocrinol (Lausanne). 2023 May 25;14:1168663. doi: 10.3389/fendo.2023.1168663 (PMC10248451; doi:10.3389/fendo.2023.1168663)
Supplement: Supplementary file 4 [file Table_3.pdf]

Table S5. Sensitivity coefficients of T4 and T3 in Liver tissue and RB tissue with respect to metabolic parameters

	k26	k34	k35	k24	k32	k33
	Liver tissue			RB tissue		
T4	-0.0026	-0.0079	-1.276E-05	-0.0176	-0.0527	-5.913E-06
T3	0.0279	-2.483E-04	-0.0413	0.255	-0.0137	-0.3078

Note: The sensitivity analysis was performed by clamping plasma *fT*4 and *fT*3 in *Body Blood* at their respective basal steady-state concentrations.
